# KSHV-encoded LANA protects the cellular replication machinery from hypoxia induced degradation

**DOI:** 10.1371/journal.ppat.1008025

**Published:** 2019-09-03

**Authors:** Rajnish Kumar Singh, Zachary L. Lamplugh, Fengchao Lang, Yan Yuan, Paul Lieberman, Jianxin You, Erle S. Robertson

**Affiliations:** 1 Department of Otorhinolaryngology-Head and Neck surgery, Perelman School of Medicine, University of Pennsylvania, Philadelphia, United States of America; 2 Department of Microbiology, Levy Building, School of Dental Medicine, University of Pennsylvania, Philadelphia, United States of America; 3 Program in Gene Regulation, The Wistar Institute, Philadelphia, United States of America; 4 Department of Microbiology, Perelman School of Medicine, University of Pennsylvania, Philadelphia, United States of America; University of South Florida, UNITED STATES

## Abstract

Kaposi’s sarcoma associated herpesvirus (KSHV), like all herpesviruses maintains lifelong persistence with its host genome in latently infected cells with only a small fraction of cells showing signatures of productive lytic replication. Modulation of cellular signaling pathways by KSHV-encoded latent antigens, and microRNAs, as well as some level of spontaneous reactivation are important requirements for establishment of viral-associated diseases. Hypoxia, a prominent characteristic of the microenvironment of cancers, can exert specific effects on cell cycle control, and DNA replication through HIF1α-dependent pathways. Furthermore, hypoxia can induce lytic replication of KSHV. The mechanism by which KSHV-encoded RNAs and antigens regulate cellular and viral replication in the hypoxic microenvironment has yet to be fully elucidated. We investigated replication-associated events in the isogenic background of KSHV positive and negative cells grown under normoxic or hypoxic conditions and discovered an indispensable role of KSHV for sustained cellular and viral replication, through protection of critical components of the replication machinery from degradation at different stages of the process. These include proteins involved in origin recognition, pre-initiation, initiation and elongation of replicating genomes. Our results demonstrate that KSHV-encoded LANA inhibits hypoxia-mediated degradation of these proteins to sustain continued replication of both host and KSHV DNA. The present study provides a new dimension to our understanding of the role of KSHV in survival and growth of viral infected cells growing under hypoxic conditions and suggests potential new strategies for targeted treatment of KSHV-associated cancer.

## Author summary

Hypoxia induces cell cycle arrest and DNA replication to minimize energy and macromolecular demands on the ATP stores of cells in this microenvironment. A select set of proteins functions as transcriptional activators in hypoxia. However, transcriptional and translational pathways are negatively regulated in response to hypoxia. This preserves ATP until the cell encounters more favorable conditions. In contrast, the genome of cancer cells replicates spontaneously under hypoxic conditions, and KSHV undergoes enhanced lytic replication. This unique feature by which KSHV genome is reactivated to induce lytic replication is important to elucidate the molecular mechanism by which cells can bypass hypoxia-mediated arrest of DNA replication in cancer cells. Here we provide data which shows that KSHV can manipulate the DNA replication machinery to support replication in hypoxia. We observed that KSHV can stabilize proteins involved in the pre-initiation, initiation and elongation steps of DNA replication. Specifically, KSHV-encoded LANA was responsible for this stabilization, and maintenance of endogenous HIF1α levels was required for stabilization of these proteins in hypoxia. Expression of LANA in KSHV negative cells confers protection of these replication proteins from hypoxia-dependent degradation, and knock-down of LANA or HIF1α showed a dramatic reduction in KSHV-dependent stabilization of replication-associated proteins in hypoxia. These data suggest a role for KSHV-encoded LANA in replication of infected cells, and provides a mechanism for sustained replication of both cellular and viral DNA in hypoxia.

## Introduction

Kaposi Sarcoma associated herpesvirus (KSHV) or Human herpesvirus 8 (HHV8) infects human endothelial cells and B-lymphocytes and is strongly associated with Kaposi sarcoma (KS), Pleural Effusion Lymphoma (PEL) and Multicentric Castleman’s Disease (MCD) [1–4]. Like other herpesviruses, KSHV maintains the viral genome as extra-chromosomal episomes in latently infected cells with only a limited number of KSHV-encoded genes expressed [5–7]. Upon successful infection and establishment of latency, cellular transformation by KSHV relies upon its ability to degrade tumor suppressors or activating pro-oncogenic factors [8–11], though immune competency of infected individual plays a critical role in pathogenesis of KSHV infection [12].

KSHV-encoded latency associated nuclear antigen (LANA) is the major factor responsible for maintaining latency as well as tethering the viral episomal DNA to host chromatin [5, 13–15]. LANA binds directly to the terminal repeats, which contains the minimal replication unit through its carboxy-terminus while binding to cellular chromatin through its amino-terminus [16, 17]. For persistent replication of the KSHV genome, LANA also recruits the clamp loader proliferating cell nuclear antigen (PCNA) to the KSHV genome [18]. Epigenetic reprogramming of the KSHV genome is another key requirement for maintaining latent infection and escaping from host immune response by switching off expression of the majority of the genes [19, 20]. Recent studies demonstrated genome wide changes in methylation patterns, as well as histone modifications throughout the steps of infection for establishment of latency or lytic reactivation post-infection [21–23]. In normoxia, the KSHV genome replicates once per cell cycle to maintain the gross copy number, and its replication is dependent on the host cellular machinery. The inhibition of KSHV replication through Geminin, an inhibitor of Cdt1 and mammalian replication confirmed the involvement of host regulatory factors in latent replication of KSHV[17]. Additionally, expression of Cdt1, rescued the replication ability of plasmids containing the KSHV minimal replicator element [17].

LANA is involved in recruiting the DNA clamp loader PCNA to mediate efficient replication and persistence of KSHV [18]. We have also previously identified and characterized another latent origin, which supports replication of plasmids ex-vivo without LANA expression in *trans* and prompted our investigation using single molecule analysis of replicated DNA (SMARD)[24, 25]. The study resulted in identification of multiple replication initiation sites within the entire KSHV genome [25]. Chromatin immuno-precipitation assays performed using anti-origin recognition complex 2 (ORC2), and LANA antibodies from nuclear extracts of cells containing plasmids RE-LBS1/2, RE-LBS1, LBS1, or RE showed an association of ORC2 with the RE region[17]. Similarly, other host *trans* factors like MCMs was shown to be associated with the replication initiation complex [25].

Hypoxia and the hypoxia inducible factor HIF1α play a critical role in pathogenesis of KSHV by modulating expression of critical KSHV-encoded genes, as well as stabilizing several KSHV-encoded proteins. Although only a few hypoxia responsive elements (HREs) within promoters of KSHV-encoded genes have been validated, there are hundreds of uncharacterized HREs present across KSHV genome. The most important HREs characterized within the promoters of KSHV genes are those within the regulatory regions of LANA, the reactivation and transcriptional activator (RTA), and the viral G-Protein coupled receptor (vGPCR) [26–28].

LANA is involved in maintenance of KSHV latency, and it promotes tumorigenic properties through either activation of oncogenic pathways or repression of apoptotic pathways [14, 29]. RTA is involved in transcriptional activation of KSHV-encoded genes and lytic replication of the KSHV genome [30, 31]. The direct involvement of hypoxia in KSHV lytic replication have been demonstrated by a number of studies which showed that HIF1α facilitated KSHV-encoded RTA-mediated reactivation by binding to LANA to upregulate RTA expression [27]. Hypoxia is also reported to enhance the viral reactivation potential of the well-known reactivating compound 12-O-tetraecanoylphorbol-13-acetate [32]. Furthermore, the role of hypoxia in maintenance of latency is also crucial, where promoters of the key latent gene cluster coding for LANA, vFLIP and vCyclin harbor hypoxia responsive elements regulated by HIF1α [28]. Hypoxia-dependent expression of vGPCR is well known for modulating expression of several metabolic genes through ROS dependent epigenetic modifications [26]. It is also important to note that vGPCR up-regulated expression of HIF1α through activation of the MAPK kinase signaling pathway through the targeting of P38 [33]. This HIF1α-vGPCR positive feedback mechanism may explain in part the elevated levels of HIF1α in KSHV infected cells. The elevated levels of HIF1α in KSHV-infected cells was shown to modulate several pathways essential for cell proliferation, apoptosis, angiogenesis and metabolic reprogramming [34].

Hypoxia is a detrimental stress to aerobic cells and a consequence of restricted blood supply in the context of *in-vivo* conditions [35]. Cessation of cell cycle progression, and DNA replication are the main adaptive response of cells to minimize their energy and macromolecular demands [36, 37]. Additionally, transcription factors specifically stabilized in hypoxia (Hypoxia inducible factors; HIFs) regulate transcription of a number of genes responsible for reprogramming cell metabolism and promote survival [38, 39]. Stabilized HIF1α is also recognized as a negative regulator of cell division and replication through its non-transcriptional associated functions [40–42]. Furthermore, HIF1α knock-down abrogates hypoxia-mediated cell cycle arrest and promotes DNA replication in the subsequent synthesis phase [42]. It is well established that KSHV infection promotes HIF1α stabilization, and paradoxically further exposure of KSHV positive cells to hypoxia induces lytic replication. [27, 32]. The differential character of replication of the KSHV genome under hypoxic conditions begs the exploration of how KSHV manipulates the replication machinery to promote latent replication under hypoxia, a non-permissive and unfavorable condition. In this study we now show that KSHV not only allows an efficient transition to S-phase by stabilizing CyclinE/CDK2, but also protects critical replication-associated proteins involved in origin recognition, initiation and elongation from hypoxia-dependent degradation. In addition, KSHV-encoded LANA in conjunction with host-encoded HIF1α is necessary for efficient replication in the hypoxic microenvironment.

## Results

### KSHV infection allows S-phase entry of cells in hypoxia by stabilizing Cyclin/Cyclin dependent Kinase 2

**Fig 1:**
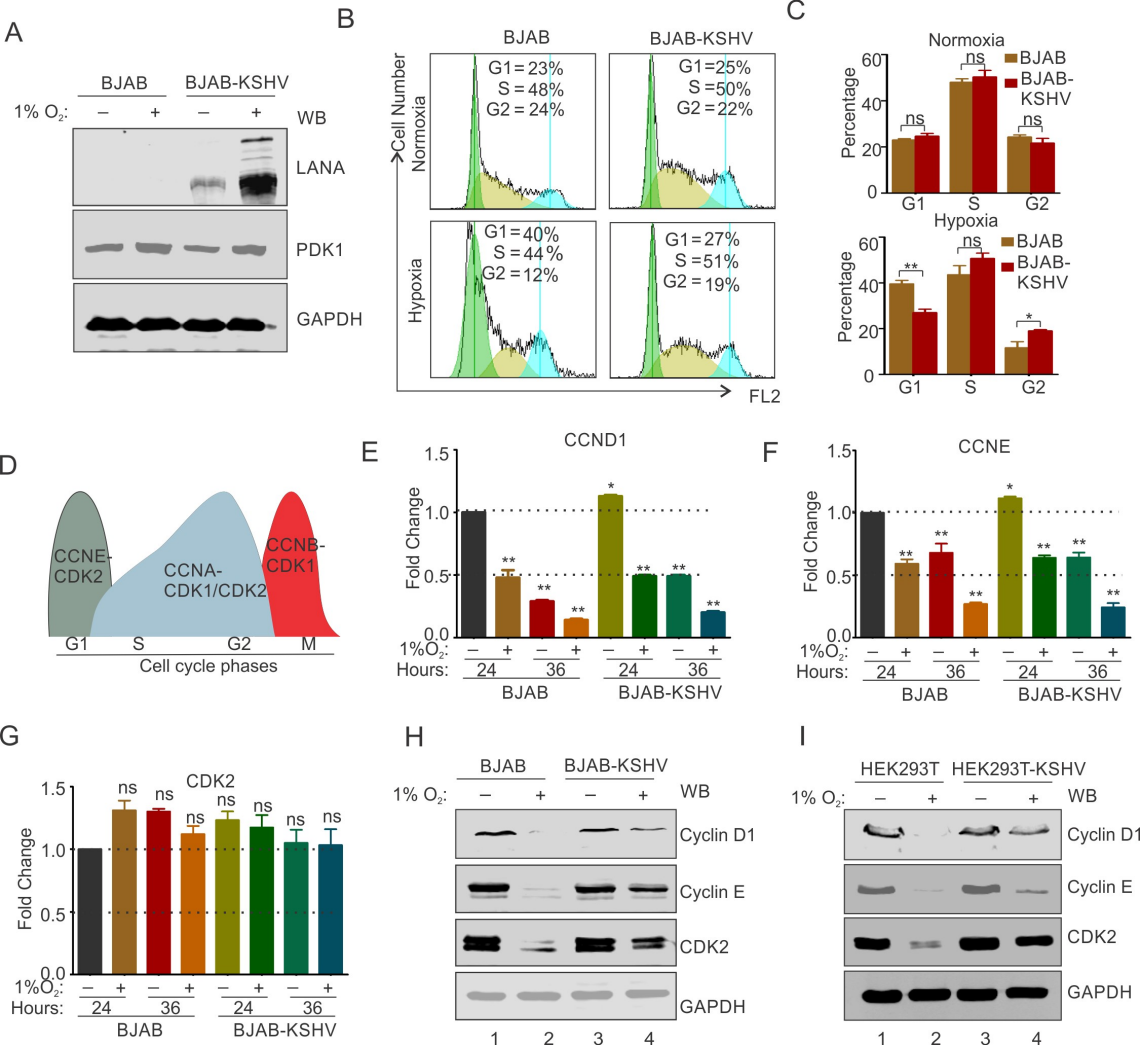
KSHV infection promotes cell proliferation and DNA replication in infected cells grown under hypoxic conditions. (A) Confirmation of induction of hypoxia by western blot for LANA and PDK1. (B) Cell cycle analysis of BJAB and BJAB-KSHV cells grown under normoxic or 1% O_2_ induced hypoxic conditions. Cells were grown for under normoxic or hypoxic conditions and stained by propidium iodide after fixation and RNase treatment. Representative image for cell cycle analysis at 24 hours are shown. (C) Barr diagram showing relative percentage of cells in various cell cycle phases in BJAB and BJAB-KSHV cells grown under normoxic or 1% O_2_ induced hypoxic conditions. (D) Schematic showing cyclin/cyclin dependent kinase pair involved in various stage of cell cycle. (E, F and G) Real-time PCR expression analysis of Cyclin D1 (CCND1), Cyclin E (CCNE) and cyclin dependent kinase 2 (CDK2) in BJAB and BJAB-KSHV cells grown under normoxic or 1% O_2_ induced hypoxic conditions. Cells were grown for 24 or 36 hours in normoxic or 1% O_2_ induced hypoxic conditions followed by RNA isolation and cDNA synthesis. Relative fold change was calculated by delta CT method taking GAPDH as endogenous control. (H) Western blot analysis for the CyclinD1, Cyclin E and CDK2 in BJAB and BJAB-KSHV cells grown under normoxic or 1% O_2_ induced hypoxic conditions. Cells were grown under normoxic or 1% O_2_ induced hypoxic conditions for 36 hours and the whole cell lysate was used for probing protein levels as indicated. GAPDH served as endogenous control. (I) Western blot analysis for the CyclinD1, Cyclin E and CDK2 in HEK293T and HEK293T-BAC16-KSHV cells grown under normoxic or 1% O_2_ induced hypoxic conditions. Cells were grown under normoxic or 1% O_2_ induced hypoxic conditions for 24 hours and the whole cell lysate was used for probing protein levels as indicated. GAPDH served as endogenous control.

Cellular adaptive response towards hypoxia includes cessation of cell cycle progression and DNA replication to minimize energy demand and to ensure cell survival. In contrast to normal cells, the KSHV genome in infected cells is known to undergo reactivation and lytic replication [27, 32]. Interestingly, both the latency associated nuclear antigen (LANA) and replication and transcriptional activator (RTA), are up-regulated under hypoxic conditions. Where the former promotes cellular proliferation and oncogenesis, and the latter is the essential mediator of lytic replication[14, 30, 43]. BJAB-KSHV cells were used to investigate the role of KSHV in modulating cellular proliferation and DNA replication events under hypoxic conditions as compared with KSHV negative BJAB cells. The two cells lines were checked for their isogenic background by short tandem repeat (STR) profiling and after thawing a similar passage number of cells was used for the experiment [26]. As expression of transcript or protein levels of HIF1α does not represent a good marker of long-term hypoxic induction [44], PDK1 levels (a transcriptional target of HIF1α) was used to demonstrate induction of hypoxia (Fig 1A). Cell cycle analysis of BJAB and BJAB-KSHV cells grown in hypoxia for different time periods clearly indicated that the presence of KSHV can facilitate the G1/S transition under hypoxic conditions (Fig 1B and 1C and S1 Fig). Hypoxia induces arrest of G1/S transition [42], and bypassing this arrest is essential for entry and subsequent DNA replication and cellular proliferation. Therefore, we hypothesized that KSHV can manipulate the cellular machinery to bypass this arrest and promote DNA replication. To investigate this, we checked the status of Cyclins (Cyclin D1 & Cyclin E) and Cyclin dependent kinase (Cdk2), associated with G1/S transition (Fig 1D) [45]. We first investigated the status of Cyclin D1, Cyclin E and Cdk2 at the transcript level in BJAB and BJAB-KSHV cells growing under normoxic or hypoxic conditions at various time points (Fig 1E, 1F and 1G and S2 Fig). The results suggested that hypoxia exerts a similar effect on the transcription expression of these genes. Briefly, an almost 50% reduction in expression of Cyclin D1 and Cyclin E was observed at 24 hours of hypoxia treatment in both BJAB and BJAB-KSHV cells. Similarly, a 75% reduction in expression of Cyclin E was observed at 36 hours of hypoxia treatment in both BJAB and BJAB-KSHV cells (Fig 1E and 1F and S2 Fig). Interestingly, the expression of Cdk2 in these cells was observed to be independent of hypoxia with no significant differences seen in expression at any time periods (Fig 1G and S2 Fig). We, therefore, hypothesized that KSHV may affect the expression of these factors at the protein level. Based on the transcript analysis of the expression, we choose to analyze the expression of these proteins after 36 hours of hypoxic treatment. Cells were grown in normoxic or hypoxic conditions for 36 hours followed by analysis by Western blot to detect the differences in the protein levels. Interestingly, we observed that levels of both Cyclin D1 and Cyclin E as well as Cdk2 were significantly reduced in KSHV negative BJAB cells (Fig 1H). The presence of KSHV in BJAB-KSHV cells had a protective effect on these proteins from hypoxia-associated degradation (Fig 1H, compare lane 2 and 4). Furthermore, to corroborate these results, we performed the same experiment in HEK293T and HEK293T-BAC16-KSHV cells. The protection of Cyclins and Cdk2 protein levels in KSHV positive HEK293T-BAC16-KSHV compared to HEK293T confirmed that KSHV was able to block hypoxia-dependent degradation to allows S-phase entry and subsequent DNA replication (Fig 1I, compare lane 2 and 4).

### KSHV infection promotes origin recognition and pre-initiation of DNA replication in hypoxia

**Fig 2:**
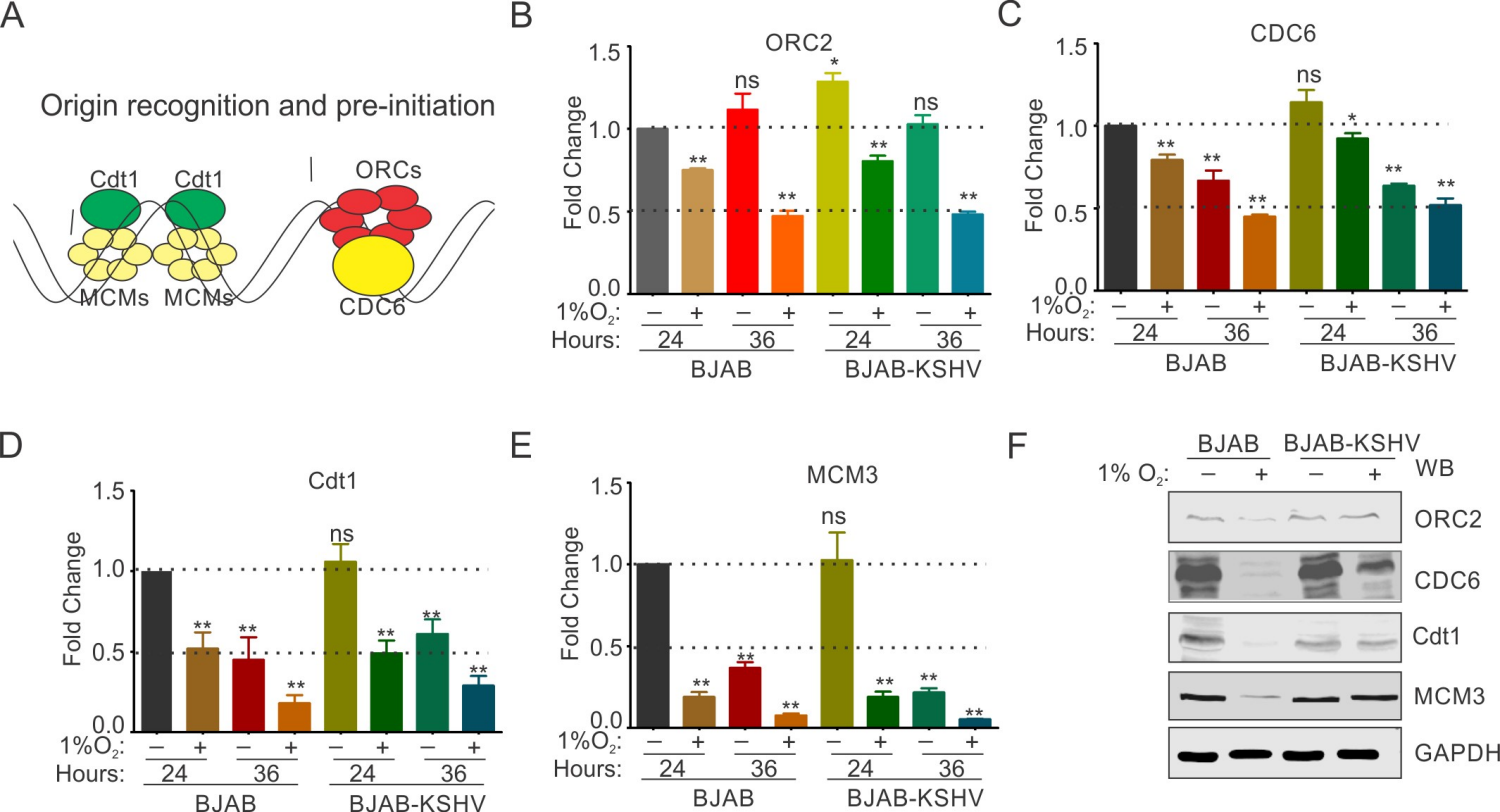
KSHV infection protects origin recognition and pre-replication proteins from hypoxia mediated degradation. (A) Schematic showing various proteins involved in origin recognition and pre-replication for the start of DNA replication. (B-E) Representative barr diagram for Real-time PCR expression analysis of ORC2, CDC6, Cdt1 and MCM3 in BJAB and BJAB-KSHV cells grown under normoxic or 1% O_2_ induced hypoxic conditions. Cells were grown for 24 or 36 hours in normoxic or 1% O_2_ induced hypoxic conditions followed by RNA isolation and cDNA synthesis. Relative fold change was calculated by delta CT method taking GAPDH as endogenous control. The experiments were performed in at least triplicates. The error bar represents standard error from the mean. Asterisk (*) represents statistically significant difference with p<0.05, **p<0.01. (F) Representative image of Western blot analysis of ORC2, CDC6, Cdt1 and MCM3 in BJAB and BJAB-KSHV cells grown under normoxic or 1% O_2_ induced hypoxic conditions. Cells were grown for 36 hours in normoxic or 1% O_2_ induced hypoxic conditions. Equal amounts of whole cell lysate were used for probing protein levels as indicated. GAPDH served as endogenous control.

Origin recognition by origin recognition complex (ORCs) proteins and formation of pre-initiation complex are the initial event of DNA replication[46]. Therefore, we investigated whether the presence of KSHV can differentially modulate origin recognition and pre-initiation steps under hypoxic conditions. A schematic showing the comprehensive list of proteins involved in origin recognition and pre-initiation of DNA replication are shown (Fig 2A). We investigated the levels of origin recognition complex proteins (ORC1-6) at both transcript and protein levels in BJAB and BJAB-KSHV cells growing under normoxic or hypoxic conditions. Additionally, levels of cell division cycle 6 (CDC6; essential component for assembly of pre-replication complex), Cdt1 (replication licensing protein essential for loading of minichromosomal maintenance proteins, MCMs), and a representative of MCMs (MCM3) were also measured at the transcript and protein levels (Fig 2A–2F). Among the ORCs, ORC1 and ORC4 appeared to be transcriptionally stable at the both 24 and 36 hours of hypoxic treatment (1%O_2_) with only a marginal down-regulation in both the BJAB and BJAB-KSHV cells (Fig 2B and S3A–S3D Fig). The expression of ORC2, ORC3 and ORC5 showed changes in expression profiles at transcript levels in both the BJAB and BJAB-KSHV cells and at both time points (24 and 36 hours). Briefly, ORC2 showed a down-regulation by nearly 20% in both BJAB and BJAB-KSHV at the end of 24 hours of hypoxic treatment. ORC2 expression was further down-regulated by nearly 50% at 36 hours of hypoxic treatment (Fig 2B). The expression of ORC3 at the transcript level showed an intermediate effect where the fold change difference was not significantly different in both BJAB and BJAB-KSHV cells at the end of 24 hours of hypoxic treatment. However, at 36 hours, expression of ORC3 was down regulated at nearly 50% in both BJAB and BJAB-KSHV cells (S3B Fig). Among the ORCs investigated, ORC5 showed the most drastic effects of hypoxia, where the fold change of ORC5 transcripts was observed to be nearly 50% down regulated at the end of 24 hours of hypoxic treatment, and by the end of 36 hours of hypoxic treatment, almost an 80% decrease was observed in both BJAB and BJAB-KSHV cells (S3D Fig). MCM3, critical for origin recognition was also dramatically down-regulated by greater than 80% in 24 hrs of hypoxia treatment and over 90% by 36 hrs (Fig 2E). The effects in BJAB-KSHV was similar but not substantially greater than in BJAB alone (Fig 2E). We hypothesized that similar to cyclins and CDK2, KSHV may influence the expression of ORCs and MCM3 at the protein level.

Western blot analyses were also performed to monitor these proteins using lysates from BJAB and BJAB-KSHV cells grown under normoxic or hypoxic conditions for 36 hours. Results clearly suggested that the presence of KSHV had a protective effect on these proteins from hypoxia-mediated degradation (Fig 2F and S3E Fig). We further corroborated these results in another KSHV positive cell lines, HEK293T-BAC16-KSHV cells compared to HEK293T cells. Similarly, protection of these proteins in HEK293T-BAC16-KSHV cells grown under hypoxic conditions confirmed that KSHV infection played a role in origin recognition by providing a level of protection for the origin recognition proteins from hypoxia-mediated degradation (S3F Fig).

### KSHV promotes increased stability of DNA replication initiation proteins in hypoxia

**Fig 3:**
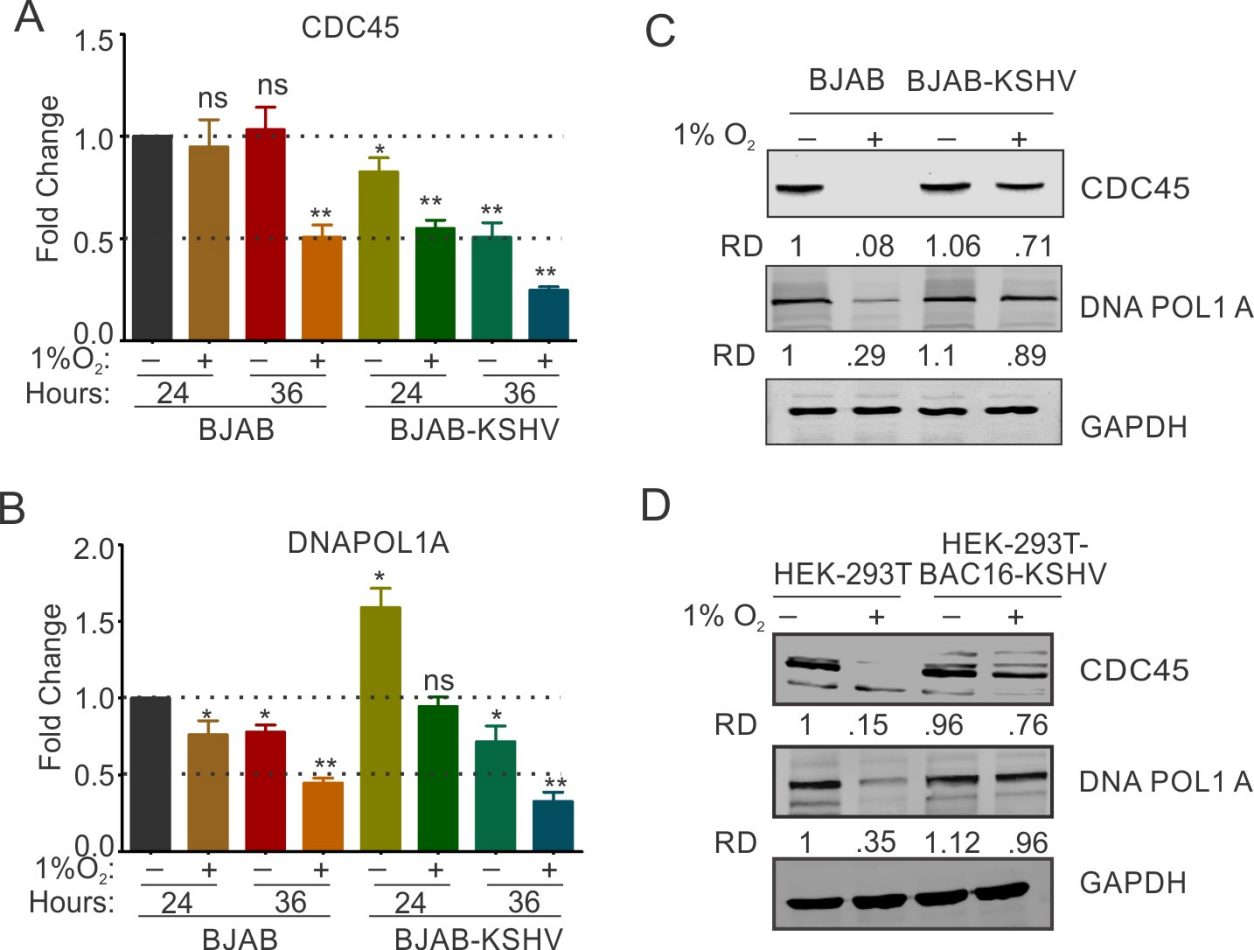
KSHV infection protects hypoxia-mediated degradation of proteins involved in replication initiation. (A and B) Real-time PCR expression analysis of CDC45 and DNAPOL1α in BJAB and BJAB-KSHV cells grown under normoxic or 1% O_2_ induced hypoxic conditions. Cells were grown for 24 or 36 hours in normoxic or 1% O_2_ induced hypoxic conditions followed by RNA isolation and cDNA synthesis. Relative fold change was calculated by delta CT method taking GAPDH as endogenous control. The experiments were performed in at least triplicates. The error bar represents standard error from the mean. Asterisk (*) represents statistically significant difference with p<0.05, **p<0.01. (C and D) Representative image of Western blot analysis of CDC45 and DNAPOL1α in BJAB and BJAB-KSHV cells grown under normoxic or 1% O_2_ induced hypoxic conditions. Cells were grown for 36 hours in normoxic or 1% O_2_ induced hypoxic conditions. Equal amount of whole cell lysate was used for probing protein levels as indicated. GAPDH served as endogenous control. (D) Representative image of Western blot analysis of CDC45 and DNAPOL1α in HEK293T and HEK293T-BAC16-KSHV cells grown under normoxic or 1% O_2_ induced hypoxic conditions. Cells were grown for 24 hours in normoxic or 1% O_2_ induced hypoxic conditions. Equal amounts of whole cell lysate were used for probing protein levels as indicated. GAPDH served as endogenous control.

Results showing KSHV-mediated protection of cell cycle, and DNA replication associated proteins from hypoxia-dependent degradation stimulated further investigation of key proteins involved in initiation of DNA replication. We investigated the levels of cell division cycle 45 (CDC45; essential for the loading of DNA polymerase 1 alpha on replication complex)[47], and DNA polymerase 1 alpha (DNA pol 1α; the rate limiting DNA polymerase with primase activity)[48] at the transcript, and protein levels in BJAB and BJAB-KSHV cells grown under normoxic or hypoxic conditions. Real-time expression analysis of these genes showed that the presence of KSHV did not provide a dramatic differential at the transcript level for CDC45 in normoxic or hypoxic conditions, but it up-regulated expression of DNAPol1 alpha to approximately 1.6-fold in normoxic condition (Fig 3A and 3B). Furthermore, at the 36 hour time point, hypoxia induced a similar level of down-regulation of transcripts by approximately 50–75% for the transcripts of both CDC45 and DNA pol 1α in BJAB and BJAB-KSHV cells grown under hypoxic conditions (Fig 3A and 3B).

Similar to the expression of genes involved in origin recognition and pe-initiation, we hypothesized that KSHV may influence the levels of these factors at the level of post-translation. Western blot analyses were performed to monitor CDC45 and DNA Pol 1α proteins in BJAB and BJAB-KSHV cells grown in normoxic or hypoxic conditions for 36 hours. The results indicated a clear protection of these replication proteins from hypoxia-mediated degradation by the presence of KSHV (Fig 3C compare lane 2 and 4). We further examined the role of KSHV as a major contributor to the inhibition of hypoxia-mediated degradation in HEK293T-BAC16-KSHV cells when compared to HEK293T cells (Fig 3D). Similarly, the levels of these proteins in HEK293T-BAC16-KSHV cells grown under hypoxic conditions were enhanced and supported a role for KSHV in origin recognition through protection of origin recognition proteins from hypoxia-mediated degradation.

### Infection of PBMCs by KSHV can rescue DNA replication-associated proteins from hypoxia-mediated degradation

**Fig 4:**
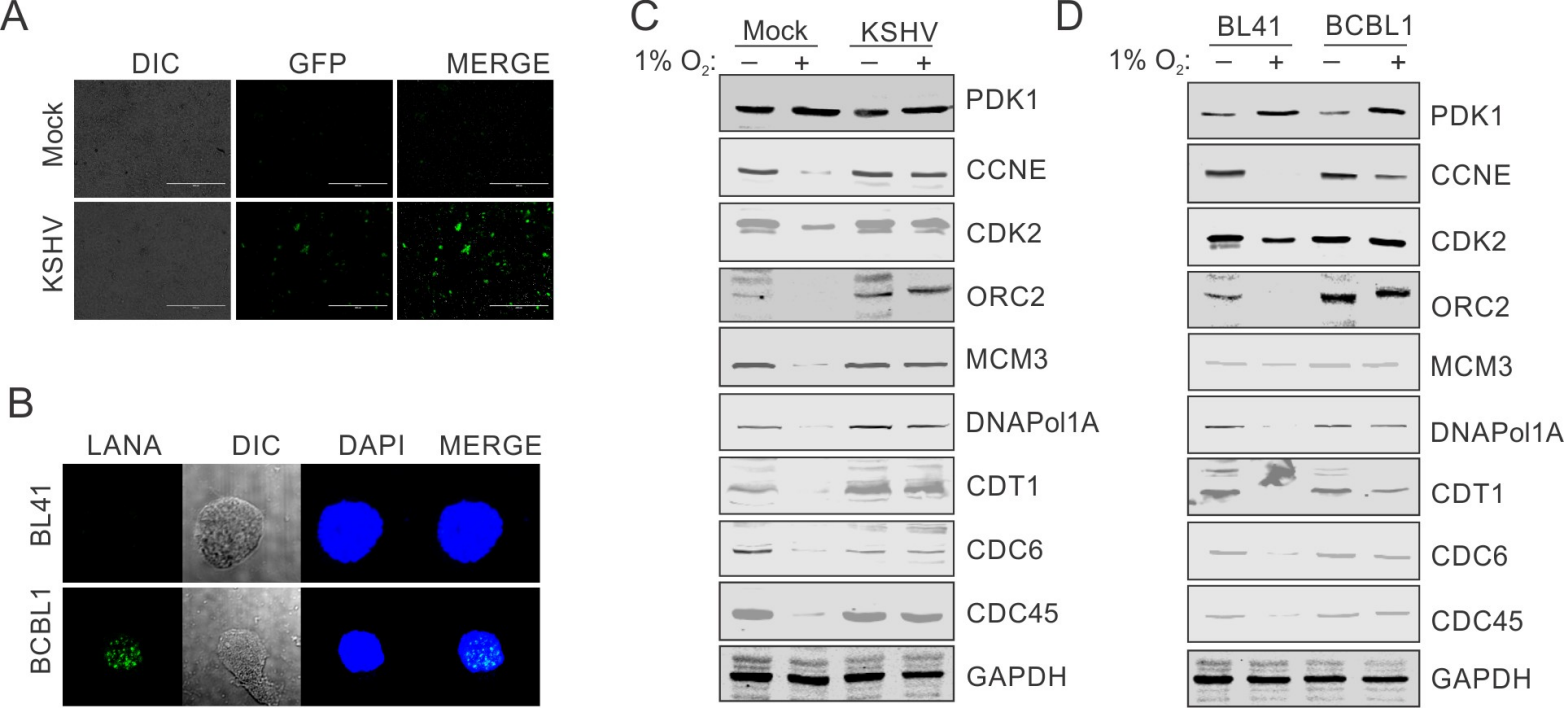
Infection of PBMCs with KSHV protects replication associated proteins from hypoxia mediated degradation. (A) Infection of PBMCs with recombinant KSHV (rKSHV) obtained from HEK293T-BAC16-KSHV. GFP expressing rKSHV virions were reactivated from HEK293T-BAC16-KSHV by growing them in the presence of TPA and butyric acid. Copy number of purified rKSHV was calculated by standard curve method using a plasmid containing KSHV genomic region. A multiplicity of infection equal to10 was used for infecting the cells in the presence of 20¼g/ml polybrene. The KSHV infection was monitored using GFP signals from the infected cells. (B) Immunostaining for LANA in KSHV negative BL41 and KSHV positive BCBL1 cells. (C) Representative image of Western blot analysis of replication associated proteins in PBMCs infected with HEK293T-BAC16-KSHV cells derived KSHV and grown under normoxic or 1% O_2_ induced hypoxic conditions. Cells were grown for 36 hours in normoxic or 1% O_2_ induced hypoxic conditions. Equal amount of whole cell lysate was used for probing protein levels as indicated. GAPDH served as endogenous control. (D) Representative image of Western blot analysis of replication associated proteins in KSHV negative BL41 and KSHV positive BCBL1cells grown under normoxic or 1% O_2_ induced hypoxic conditions. Cells were grown for 36 hours in normoxic or 1% O_2_ induced hypoxic conditions. Equal amounts of whole cell lysate were used for probing protein levels as indicated. GAPDH served as endogenous control.

To further rule out any possible role due to differences in the source of BJAB/BJAB-KSHV or HEK293T/ HEK293T-BAC16-KSHV cells or their passage numbers, we infected PBMCs with KSHV generated from HEK293T-BAC16-KSHV. We infected PBMCs with purified KSHV at a multiplicity of infection equal to 10. Initially, the infected cells were grown under normoxic conditions for 24 hours to allow for the expression of KSHV-encoded genes in the infected cells. KSHV infection of PBMCs was confirmed by GFP signals (for infection with KSHV generated from HEK293T-BAC16-KSHV) (Fig 4A). The mock control or infected PBMCs were then incubated under hypoxic conditions for another 24 hours. The induction of hypoxia was confirmed by western blot against PDK1. The status of the proteins examined above were monitored by western blot. Similar to the results seen in BJAB or HEK293T cells, a significant decrease in the level of all the proteins investigated (CCNE, CDK2, ORC2, MCM3, CDC6, Cdt1, CDC45 and DNAPol1A) was observed (Fig 4C). As expected, KSHV infected PBMCs grown under hypoxic conditions was able to rescue these proteins from hypoxia-mediated degradation (Fig 4C). These results strongly supported a role for KSHV in the rescue of DNA replication-associated proteins from hypoxia-mediated degradation. In another approach, levels of these proteins in KSHV negative BL41 were compared with KSHV positive BCBL1 cells. The cell lines were confirmed for the absence or presence of KSHV by immune staining against LANA protein (Fig 4B). The cells were grown under hypoxic conditions followed by analysis of PDK1 levels for the confirmation of induction of hypoxia. The comparative analysis of replication associated proteins further confirmed the protection potential of KSHV for replication associated proteins from hypoxia-mediated degradation (Fig 4D).

### KSHV-encoded LANA protects DNA replication-associated proteins from hypoxia-mediated degradation

**Fig 5:**
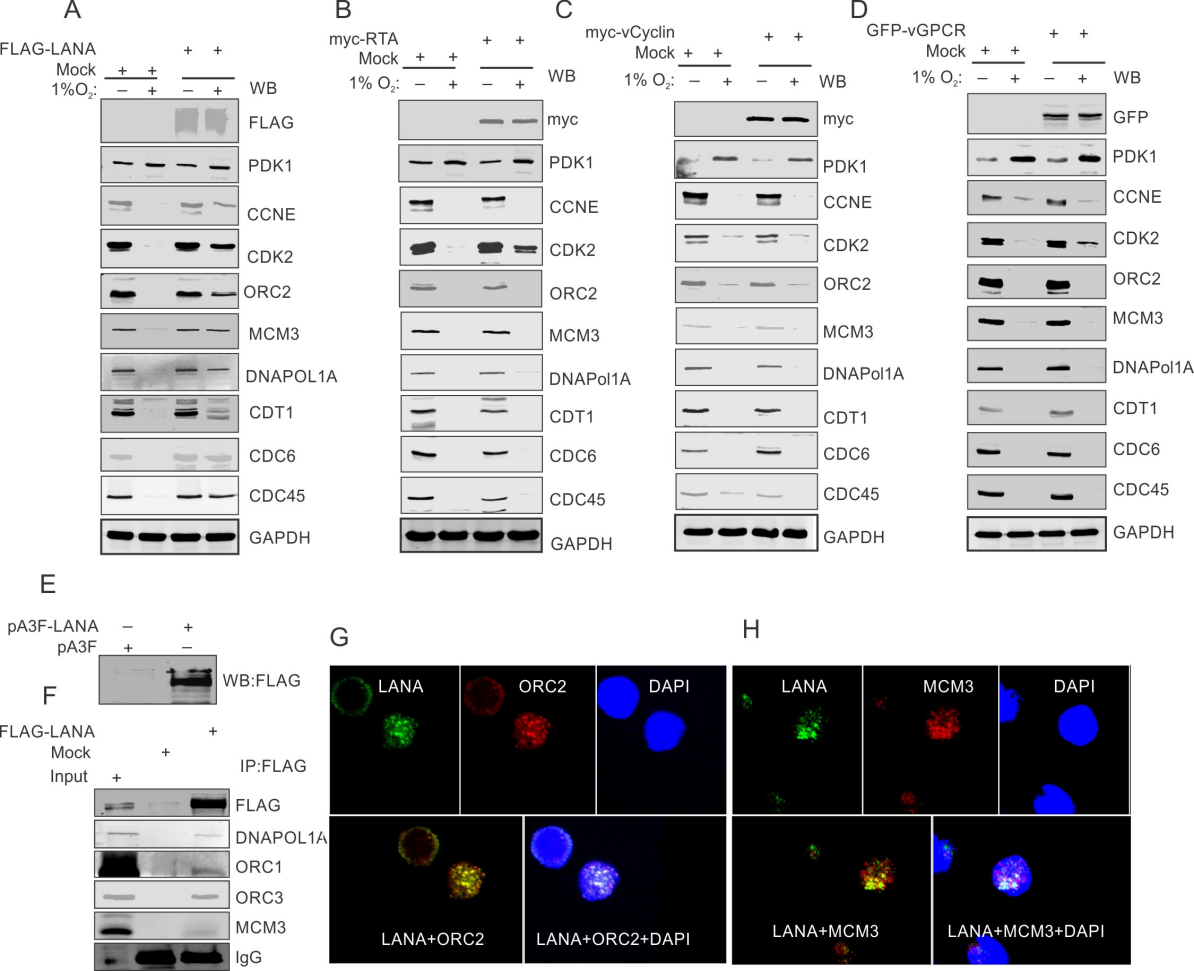
KSHV encoded LANA protects hypoxia-mediated degradation of proteins involved in DNA replication. (A) Mock or FLAG-tagged LANA was ectopically expressed in HEK293T cells followed by growing these cells in normoxic or 1% O_2_ induced hypoxic conditions. Ectopic expression of LANA was confirmed by immunoblot against FLAG. Representative image for immunoblot of replication associated proteins are given showing LANA mediated protection of these proteins from hypoxia dependent degradation. GAPDH served as loading control. (B) Mock or myc-tagged RTA was ectopically expressed in HEK293T cells followed by growing these cells in normoxic or 1% O_2_ induced hypoxic conditions. Ectopic expression of RTA was confirmed by immunoblot against myc. Representative image for immunoblot of replication associated proteins are given showing that RTA is incapable of protection for these proteins from hypoxia dependent degradation. GAPDH served as loading control. (C) Mock or GFP-tagged vCyclin was ectopically expressed in HEK293T cells followed by growing these cells in normoxic or 1% O_2_ induced hypoxic conditions. Ectopic expression of vCyclin was confirmed by immunoblot against GFP. Representative image for immunoblot of replication-associated proteins are given showing vCyclin is incapable of protection for these proteins from hypoxia-dependent degradation. GAPDH served as a loading control. (D) Mock or GFP-vGPCR was ectopically expressed in HEK293T cells followed by growing these cells in normoxic or 1% O_2_ induced hypoxic conditions. Ectopic expression of vGPCR was confirmed by immunoblot against GFP. Representative image for immunoblot of replication associated proteins shows that vGPCR provides no detectable protection for these proteins from hypoxia-dependent degradation. GAPDH served as loading control. (E, F, G and H) KSHV-encoded LANA interacts with replication associated proteins in hypoxic conditions. (E) Ectopic expression of FLAG-LANA. (F) Immuno-precipitation by FLAG (LANA) and western blot for FLAG, DNAPOL1A, ORC1, ORC3 and MCM3. The immune-precipitated samples were run against 2.5% input. (G) Representative image of immuno-fluorescence showing LANA interaction with ORC2 and MCM3.

Latently infected KSHV positive cells predominantly express only a limited set of KSHV-encoded proteins. Furthermore, hypoxia is well known to induce expression of other KSHV-encoded genes. The latency associated nuclear antigen (LANA), replication and transcriptional activator (RTA), viral G-Protein coupled receptor (vGPCR) and viral cyclin (vCyclin), are well-established antigens being expressed either in latency or during hypoxic conditions [26–28]. We hypothesized that one, or a combination of these proteins will be responsible for rescuing the DNA replication-associated proteins from hypoxia-mediated degradation. To identify which of the KSHV-encoded protein was responsible for rescuing DNA replication-associated proteins from hypoxia-mediated degradation, we individually expressed these proteins in HEK293T cells along with mock transfection as control. Transfected cells were allowed to grow under normoxic or hypoxic conditions. The expression of KSHV-encoded antigens was confirmed using western blots with antibodies against the epitope tag fused to these proteins (Fig 5A–5D, top panel). The induction of hypoxia was confirmed by western blot analysis of PDK1 (Fig 5A–5D, second panel at the top).

Interestingly, analysis of the levels of proteins involved in DNA replication revealed that ectopic expression of KSHV-encoded LANA efficiently rescued these proteins from hypoxia-mediated degradation. Representative blots for the proteins that clearly showed that degradation occurred in hypoxia are shown (Fig 5A). Importantly, the expression of other KSHV-encoded antigens such as RTA, vGPCR or vCyclin showed little or no ability to protect these proteins from hypoxia-mediated degradation (Fig 5B–5D).

We then wanted to investigate the mechanism of how KSHV-encoded LANA protected these proteins from hypoxia-mediated degradation. KSHV-encoded LANA can interact with a number of replication-associated proteins such as ORC2 and MCM3 under normoxic conditions[49]. Therefore, we hypothesized that LANA may interact with these proteins to block their degradation in hypoxia by interfering with their ubiquitination. To demonstrate their interaction, FLAG tagged LANA was expressed in HEK293T cells (Fig 5E). Cells were grown under hypoxic conditions followed by immuno-precipitation assays using anti-FLAG antibodies. The results indicated that LANA associated with the select set of replication-associated proteins when grown under hypoxic conditions (Fig 5F). Further validation of these associations in cellular complexes was examined using immuno-fluorescence assays taking ORC2 and MCM3 as representative proteins. The results showed that LANA and ORC2 or LANA and MCM3 co-localized under hypoxic conditions which corroborated their association in cellular replication compartments (Fig 5G and 5H).

### Knock down of LANA in KSHV positive cells resulted in a loss of ability to rescue replication proteins from hypoxia-dependent degradation

**Fig 6:**
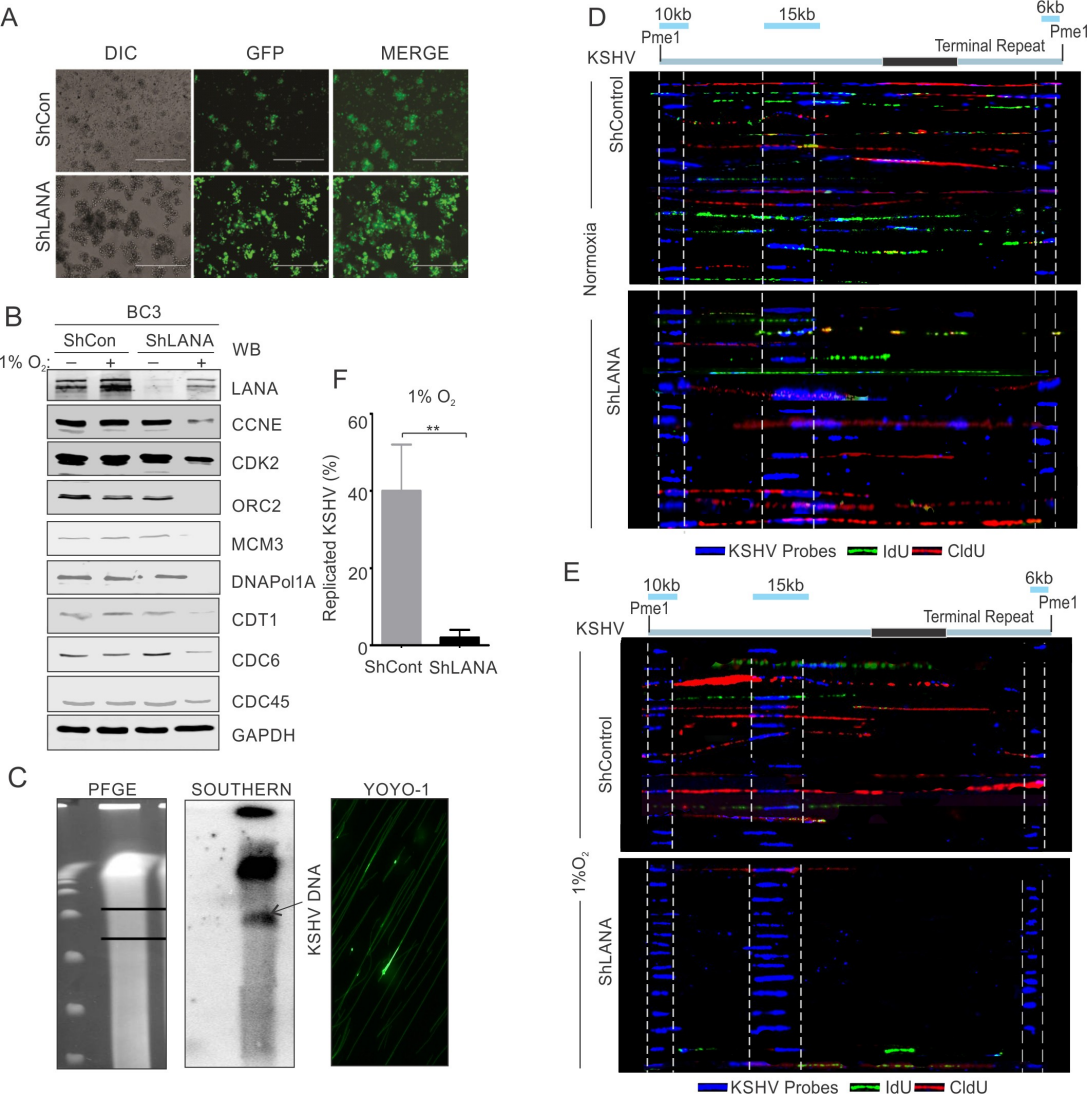
LANA knock down KSHV positive cells are replication incompetent when grown under hypoxic conditions. (A) Representative image for GFP fluorescence in BC3 cells confirming generation of ShControl and ShLANA cells. (B, Top panel) western blot analysis for LANA in BC3-ShControl and BC3-ShLANA cells. (B, Other panels) Representative image for immunoblot of replication associated proteins are given showing knock-down of LANA is incapable of protection for these proteins from hypoxia dependent degradation. GAPDH served as loading control. (C) Representative image for pulse field gel electrophoresis, Southern blot analysis and YOYO-1 staining for IdU/CldU pulsed cells. BC3 ShControl or ShLANA Cells were grown under normoxic or hypoxic conditions for 36 hours followed by collecting the cells by centrifugation. Cells were resuspended in complete medium containing 1st pulse (CldU) and grown under hypoxic conditions for 4 hours. At the end of pulsing, cells were collected by centrifugation and again resuspended in fresh medium containing 2^nd^ pulse (IdU) followed by growing the cells in hypoxic conditions for another 4 hours. Cells were collected by centrifugation, mixed with InCert agarose and used to make plugs. Plugs were digested with Proteinase K and digested with Pme1. The Pme1 digested plugs were run on PFGE. The DNA from PFGE was transferred to nylon membrane. TR elements of KSHV DNA was used to create a radiolabeled probe for detecting KSHV genome by southern blot. For YOYO-1 staining and spreading of DNA on silanized slides, the region in PFGE corresponding to KSHV DNA (Southern blot) was recovered and digested with Gelase. DNA was stained with YOYO-1 dye overnight and spread over silanized slides. (D and E) Probe hybridization and IdU/CldU immuno-staining. The DNA on silanized slides was probed with three KSHV derived biotinylated probes (6kb KSHV genomic fragment; co-ordinate 26937–33194, 10kb KSHV genomic fragment; co-ordinate 36883–47193 and 15kb KSHV genomic fragment; co-ordinate 85820–100784). KSHV probes were detected using Alexa 350 conjugated secondary antibodies against biotin. IdU and CldU incorporation were detected using Alexa 488 and Alexa 595 conjugated secondary antibodies against anti-IdU (Green) and anti-CldU (Red) antibodies respectively. A representative image showing single molecule analysis of replicated KSHV DNA genome from BC3 ShControl or ShLANA cells were grown under hypoxic conditions. 15 KSHV molecules captured from random slides are shown. (F) Barr diagram showing non-replicated KSHV DNA in BC3 ShLANA compared to BC3 ShControl cells grown under 1% O2 induced hypoxic conditions.

To further support the role of LANA in protecting DNA replication-associated proteins from hypoxia-mediated degradation, we investigated whether knock down of KSHV-encoded LANA in KSHV infected cells resulted in a loss of protection potential due to the presence of KSHV. The lentivirus-mediated knock down of KSHV-encoded LANA in BC3 cells was previously described[50]. BC3-ShControl or BC3-ShLANA cells were monitored for GFP fluorescence as well as the down-regulated expression of LANA at the protein levels (Fig 6A and 6B, top panel). BC3-ShControl or BC3-ShLANA cells were incubated under normoxic or hypoxic conditions for 36 hours. This was followed by investigation of the levels of a representative set of proteins involved in DNA replication. Western blot analysis for the proteins in these cells confirmed that LANA was a crucial viral antigen required for inhibition of degradation of these proteins under hypoxic conditions (Fig 6B). Briefly, the levels of CCNE, CDK2, ORC2, MCM3, CDC6, Cdt1, CDC45 and DNAPol1A were investigated. As expected, BC3-ShControl cells showed almost similar levels of these proteins independent of whether grown under normoxic or hypoxic conditions (Fig 6B; lane 1 compared to lane 2). However, BC3-ShLANA cells grown under hypoxic conditions showed a relatively lower level of these proteins under hypoxic conditions when compared to normoxic conditions (Fig 6B, lane 2 compared to lane 4). Further, the role of LANA knock-down was validated by single molecule analysis of replicated DNA (SMARD). Analysis for replicated vs non-replicated KSHV was performed by pulsing the BC3-ShControl or BC3-ShLANA cells, and visualizing KSHV DNA using KSHV specific probes while replicated DNA was visualized by immuno-staining against IdU/CldU (Fig 6C–6E). A significantly low level of replication was observed in BC3-ShLANA cells compared to BC3-ShControl cells grown under hypoxic conditions (see lower compartment, Fig 6E). Even under the normoxic conditions, knock down of LANA also had a negative effect on KSHV replication, but significantly less compared to hypoxic conditions (Fig 6D). These experiments clearly showed that the differences seen in the stabilization of replication associated proteins in hypoxic conditions was mainly at the protein level and not at the transcript level. We further showed that, that this occurred through inhibition of the ubiquitin-mediated proteosomal degradation system which is targeted by KSHV-encoded LANA. Cells grown in hypoxic condition with medium containing proteosomal inhibitor MG132 was compared with cells grown under normoxic or hypoxic conditions. The results strongly suggested that the presence of MG132 had a protective effect on these proteins from hypoxia-mediated degradation (S4A Fig). Also, a representative protein CDC6 was used to confirm role of LANA in inhibition of proteosomal degradation in hypoxic conditions. Cells expressing mock or LANA were grown under hypoxic conditions (with or without MG132) followed by immuno-precipitation and western blot with ubiquitin antibody. The results showed that presence of LANA significantly reduces ubiquitination under hypoxic conditions (S4B Fig, compare lane 2 and 4) and suggested that LANA is likely inhibiting the activity of one of the cellular E3-ubiquitin ligase.

### HIF1α is required for rescue of hypoxia-dependent degradation of DNA replication-associated proteins

**Fig 7:**
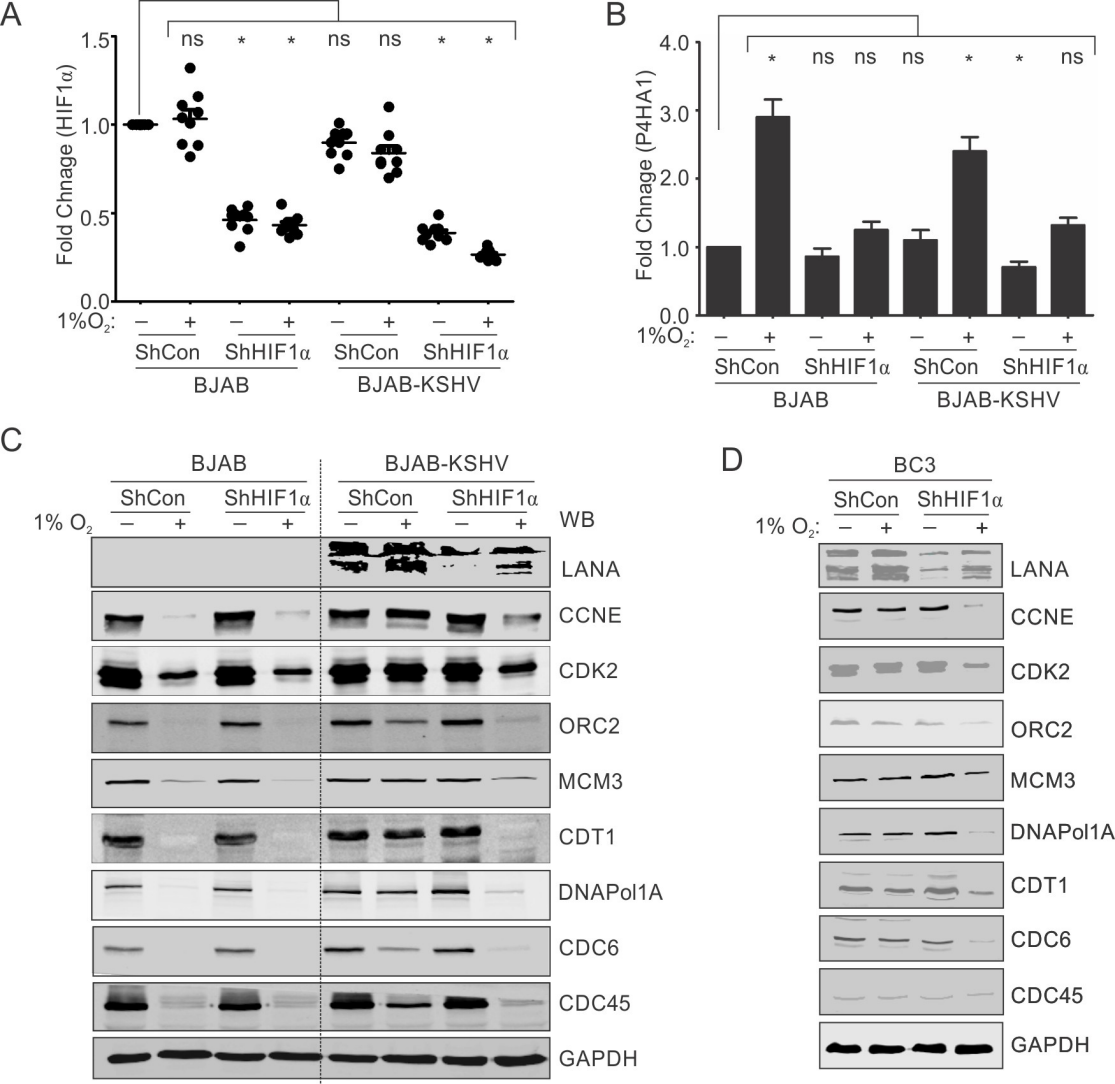
HIF1α is necessary for LANA-mediated protection of replication associated proteins from hypoxia-dependent degradation. (A) BJAB and BJAB-KSHV cells were transfected with plasmid vector encoding ShControl or ShHIF1α. The HIF1α knock-down was confirmed by real-time PCR. Briefly, 48 hours post transfection, total RNA was isolated and cDNA was synthesized. The cDNA was used for real-time PCR. (B) HIF1α knock down was confirmed by calculating fold change expression of a known HIF1α target P4HA1 by growing these cells under hypoxic conditions. The experiments were performed at least in triplicate. The error bar represents standard error from the mean. Asterisk represents statistically significant difference. (C) Representative image for Western blot analysis of replication-associated proteins in BJAB-ShControl, BJAB-ShHIF1α, BJAB-KSHV-ShControl and BJAB-KSHV-ShHIF1α cells grown under normoxic or 1% O_2_ induced hypoxic conditions. The ShControl or ShHIF1α cells were grown under normoxic or 1% O_2_ induced hypoxic conditions for 24 hours. Equal amounts of protein were used to probe with indicated antibodies. (D) Representative image for Western blot analysis of replication associate proteins in BC3-ShControl, BC3-ShHIF1α cells grown under normoxic or 1% O_2_ induced hypoxic conditions. The ShControl or ShHIF1α expressing cells were grown under normoxic or 1% O_2_ induced hypoxic conditions for 24 hours. Equal amounts of protein were used to probe with indicated antibodies. GAPDH served as loading control for panels C and D.

HIF1α is a major cellular regulator which plays a critical role in KSHV-mediated oncogenesis and is known to interact at the transcriptional and post-transcriptional levels with KSHV factors to promote the cancer phenotype. Also, HIF1α is required for upregulation of LANA during growth of KSHV positive cells in hypoxia [28]. We wanted to investigate the role of HIF1α in hypoxia-mediated degradation of DNA replication-associated proteins in KSHV negative background or their protection in KSHV-positive background. To study the role of HIF1α, we transduced both BJAB and BJAB-KSHV cells with plasmid vectors containing ShControl or ShHIF1α. Knock down of HIF1α was confirmed by real-time PCR by monitoring the HIF1α transcripts in these cells grown under normoxic or hypoxic conditions (Fig 7A). The effect of HIF1α knock down was further confirmed by investigating expression of P4HA1, a known target of HIF1α after these cells were grown under normoxic or hypoxic conditions (Fig 7B). As expected, the fold change expression of P4HA1 in both BJAB and BJAB-KSHV cells transfected with a ShHIF1α construct was significantly less when compared to the cells containing the ShControl construct under similar conditions (Fig 7B). Upon, confirmation of HIF1α knock down in both BJAB and BJAB-KSHV cells, analysis of the levels of DNA replication- associated proteins was analyzed in cells grown under normoxic or hypoxic conditions. As expected, we observed an almost complete loss of all proteins investigated in BJAB cells transfected with either ShControl or ShHIF1α constructs. Notably, BJAB-KSHV cells showed rescue of these proteins in hypoxia when transfected with ShControl plasmid. Interestingly, knock down of HIF1α in BJAB-KSHV cells showed that the ability of KSHV to protect these proteins from hypoxia-mediated degradation was severely compromised (Fig 7C). These results suggested that HIF1α contributes to the effects of KSHV-encoded LANA-mediated rescue of the replication-associated proteins from hypoxia-mediated degradation. The observation of HIF1α knock down dependent degradation of replication associated proteins in hypoxia were further validated in naturally infected KSHV positive BC3 cells. Generation and characterization of BC3-ShControl and BC3-ShHIF1α were described earlier[26]. BC3-ShControl and BC3-ShHIF1α cells were grown under normoxic or hypoxic conditions followed by investigation of the replication-associated proteins. As expected, the BC3-ShControl cells showed protection from degradation for all the studied proteins in hypoxia while BC3-ShHIF1α cells were unable to protect these proteins under hypoxic conditions (Fig 7D). The results clearly suggest that HIF1α dependent transactivation of LANA is required for rescue of these proteins from degradation in hypoxic conditions as LANA levels were substantially reduced in the BC3 shHIF1α cells. Finally, we investigated whether KSHV reactivation was induced in the hypoxic conditions. We incubated BJAB-KSHV as well as naturally infected BC3 cells under normoxic and hypoxic conditions and estimated the relative yield of KSHV in the extracellular medium. The yield was compared with the cells grown in normoxic conditions for the maximum time period used for hypoxic induction. As expected, the results clearly showed that hypoxia induced viral reactivation (S4C Fig).

## Discussion

Oncogenic herpesviridae signatures are frequently found in blood and tissue samples of the world’s population with high representations in cancer patients [51, 52]. Pathogenesis due to herpesviridae infection is also a consequence of multi-factorial events, which depends on immune status, as well as genetic heterogeneity of infected individuals [12, 53, 54]. KSHV, a large double stranded DNA containing virus was identified in the late 20^th^ century and its infection correlated strongly with the incidences of KS, PEL, MCD and KSHV inflammatory cytokine syndrome (KICS)[3, 55, 56]. During latent infection, epigenetic modification of the KSHV genome allows expression of only a limited number of KSHV encoded genes such as LANA, vFLIP, vCyclin and certain viral interferon regulatory factors [7, 22, 57].

LANA is a necessary factor for tethering of KSHV episomes to the host genome and also functions as a master regulator of latency [14, 58]. LANA can support latent replication through binding to the terminal repeats, as well as supporting hypoxia-mediated lytic replication by cooperating with HIF1α to up-regulate expression of the RTA[27]. Several viral gene products, including the KSHV LANA, vGPCR and vIRF-3 proteins, have been shown to influence HIF1α to function directly through protein-protein interaction or indirectly by enhancing transcriptional or post-transcriptional events [59–61]. LANA augmented HIF-1α stabilization by degrading VHL in the EC_5_S ubiquitin complex [9], and HIF-1α protein levels is higher in KSHV-positive PEL lines when compared to KSHV-negative cells. Additionally, expression studies showed that HIF-1α is enhanced by LANA, and that LANA stimulated the nuclear accumulation of HIF-1α[62, 63].

Notably, expression of vGPCR into NIH 3T3 mouse fibroblast cells resulted in activation of MEK and p38 signaling cascades, leading to direct phosphorylation of HIF-1α, and thus subsequent increases in HIF-1α transcriptional activity [33]. Furthermore, another latent nuclear antigen-2 (LANA-2) gene of KSHV, more commonly known as viral interferon regulatory factor 3 (vIRF-3), was implicated in the stabilization of HIF-1α, in addition to its oncogenic role of p53 inhibition [64]. Under normoxic conditions, vIRF-3 binds to the bHLH domain of HIF-1α and inhibits the breakdown of HIF-1α, which does not have an impact on its dimerization capability, but further enhanced the nuclear localization and transcriptional activity of HIF-1α [64]. The well characterized KSHV genetic loci influenced by hypoxia include open reading frames for LANA, RTA and vGPCR [26–28]. KSHV-encoded vGPCR is a constitutively active homolog of cellular GPCR and a bona-fide oncogene[65]. It can activate expression of HIF1α and in turn acts on MAP kinase pathways to enhance tumor development and angiogenesis [33]. The KSHV-encoded LANA can interact directly with HIF1α to activate expression of KSHV-encoded reactivation and transcriptional activator RTA [27]. We have recently observed that KSHV-encoded vGPCR is itself under the control of HIF1α, which activates its expression through its action at HREs within the vGPCR promoter to upregulate its expression [26]. Critically, hypoxia-dependent activation of vGPCR can potentially reprogram the metabolism of infected cells globally through generation of reactive oxygen species as well as targeting expression of DNA methyl transferases. In fact, hypoxia can work globally on the KSHV genome to modulate transcription of KSHV-encoded genes [26].

Hypoxia, in general exerts an arrest of cell cycle and DNA replication through activation of HIF1α, ATM, p53 and p21 dependent pathways as well as suppression of Myc dependent transcriptional cascade [41, 42, 66]. Furthermore, stabilized HIF1α binds efficiently with minichromosomal maintenance proteins (MCMs) to keep them inactive and keep the replication machinery in a dormant stage as a direct inhibition of DNA replication during hypoxic conditions [67]. Despite these negative regulations, KSHV infected cells bypass the G1/S transition to enter S-phase and allows productive replication (reactivation) of KSHV through yet undefined mechanisms [27, 32]. In this study, we investigated how KSHV manipulated hypoxia-mediated inhibition of DNA replication to drive replication when grown in this non-permissive and non-favorable condition. As the infection-based experiments using purified KSHV pose restrictions due to low and variable infection rates, as well as epigenetic reprogramming of the KSHV genome after entering the cells which lead to variable expression of KSHV-encoded genes, we compared the differential between BJAB and BJAB-KSHV cells, or HEK293T and HEK293T-BAC16-KSHV cells. Though, these cells are not naturally infected by KSHV, the presence of the complete KSHV genome confers to these cells the characteristics of latently infected cells. Comparative studies between these cells grown under normoxic or hypoxic conditions allowed for observation of the role of KSHV in protection of essential proteins required for G1/S transition (through stabilized Cyclins/CDK2), origin recognition (ORC1-5), Pre-initiation, initiation and elongation associated proteins (for example MCM, CDCs, and DNAPol1A). These results were replicated in PBMCs after infection with purified KSHV further supporting the role of KSHV in protecting cell cycle and DNA replication-associated proteins from hypoxia-mediated degradation. Interestingly, these differences were mainly observed at the level of proteins and not at the levels of transcript. The effect of hypoxia at the levels of transcript was similar in both KSHV negative and positive conditions suggesting that the stabilization was at the post translational stage.

**Fig 8:**
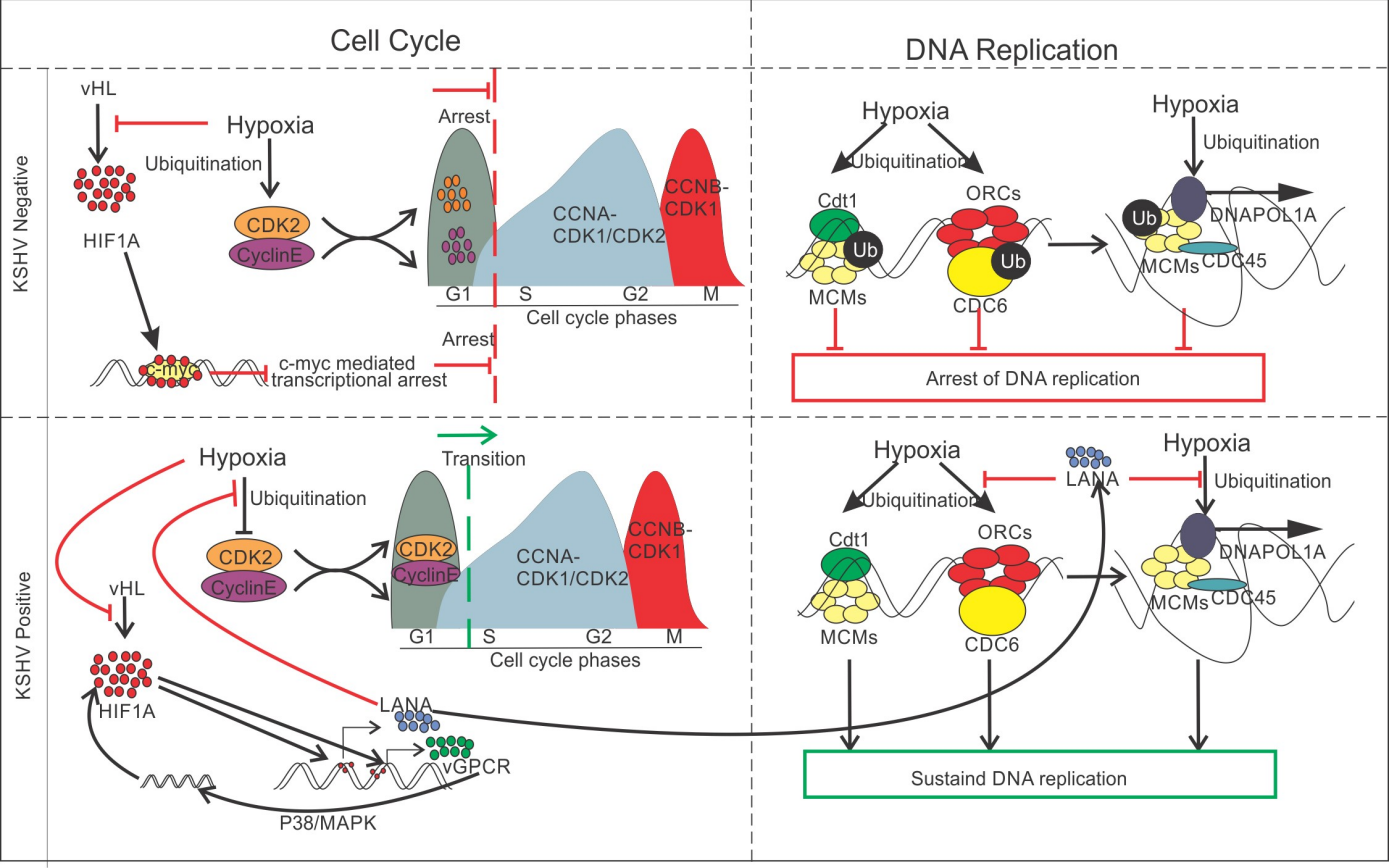
Schematic of KSHV-encoded LANA-mediated protection of replication associated proteins from hypoxia-dependent degradation. Hypoxia up-regulates expression of KSHV-encoded LANA, RTA and vGPCR by HIF1α-mediated transactivation of HREs within the promoter region. LANA cumulatively works with hypoxia to maintain a high level of HIF1α in KSHV positive cells through degradation of the tumor suppressor VHL. Additionally, LANA-HIF1α interaction facilitates the up-regulation of RTA to promote KSHV lytic replication. Moreover, hypoxia induces up-regulation of KSHV-encoded vGPCR by HIF1α-transactivation through HREs within vGPCR promoter to maintain high HIF1α level in a feedback manner. LANA interacts with several cell cycle proteins (Cyclins and CDK2) and replication-associated proteins (such as ORCs, MCMs, CDCs and DNAPOL1A). This leads to their protection from hypoxia dependent degradation. This protection is likely through its ability to interfere with ubiquitination of these proteins under hypoxic conditions.

The indispensable role of KSHV-encoded LANA in protecting these proteins from hypoxia-mediated degradation added a new function to the list of activities related to this multi-functional bonafide oncoprotein. The role was further confirmed by monitoring levels of ubiquitination of replication associated proteins in LANA expressing cells grown under hypoxic condition, which was significantly less compared to mock expressing cells grown under similar conditions. Importantly, the role of HIF1α in stabilization of these proteins provides additional clues as to why KSHV induced expression of this protein in infected cells. Combining the knowledge of HIF1α-mediated activation of KSHV-encoded antigens, and feedback regulation of HIF1α-vGPCR-HIF1α for sustained high levels of HIF1α as well as upregulation of LANA/RTA by HIF1α provides a more comprehensive strategy employed by KSHV to maintain continuous replication in hypoxia. It also provides additional information as to the mechanism by which KSHV is reactivated in non-permissive and non-favorable hypoxic conditions (Fig 8). Though, the slight stabilization of CDK2 protein under hypoxic conditions due to expression of RTA or vGPCR remain unclear, it is a matter for further investigation if these antigens do contribute by playing a role in transcriptional activation or stabilization through other strategies.

A number of questions remain unexplored in this study such as the regulatory proteins involved, mainly ubiquitin ligases that are likely targeted by LANA to mediate the stabilization of replication-associated proteins. LANA was shown to form complexes with replication-associated proteins in the replication compartments to regulate their activities by enhancing their stability in hypoxia, which also requires HIF1α activities. Also, the domain of LANA responsible for protecting cell cycle and replication-associated proteins from hypoxia dependent degradation would also be important to identify. Another factor associated with hypoxia, and responsible for G1/S arrest or replication stress is the shortage of energy in the form ATP, where molecular oxygen is an essential component for generation of energy through oxidative phosphorylation[68]. Shortage of ATP in hypoxic conditions pose a direct mechanism for termination of replication elongation. Identifying the mechanism by which these cells are able to manage this energy deficit for sustained replication in hypoxic condition would be another interesting topic to explore. Further, studies are ongoing to identify the different E3-ubiquitin ligases targeted by LANA in hypoxia to provide a more comprehensive picture of the molecular mechanism and to design targeted therapeutic strategies for intervention against KSHV-associated pathologies. Further, as sustained transcription of viral and host genes are also a pre-requisite for proper productive replication and maturation of virus upon reactivation, it would be interesting to investigate the role of KSHV infection on transcriptional stabilization under hypoxic conditions. Additionally, a comparative analysis of epigenetic reprogramming of KSHV genome under hypoxic conditions, which leads to release of repressors of lytic replication would be other area of exploration. These studies further elucidate the mechanism through which modulation of viral and host physiology under hypoxic conditions is regulated by KSHV.

## Material and methods

### Ethics statement

Peripheral blood mononuclear cells (PBMCs) from undefined and healthy donors were obtained from the Human Immunology Core (HIC) of University of Pennsylvania. The Core maintains approved protocols of Institutional Review Board (IRB) in which a Declaration of Helsinki protocols were followed, and each donor/patient gave written, informed consent.

### Plasmid constructs, cell culture, transfection, hypoxic induction and MG132 treatment

KSHV-negative BJAB cells were obtained from Elliot Kieff (Harvard Medical School, Boston, MA) and BJAB cells stably transfected with KSHV (BJAB-KSHV) were obtained from Michael Lagunoff (University of Washington, Seattle, WA). KSHV-positive body cavity lymphoma-derived BC3 and BCBL1 cells were obtained from the American type culture collection (ATCC) (Manassas, VA). BC3-ShControl and BC3-ShHIF1α cells were generated by lentivirus mediated transduction as described earlier [26]. BJAB, BJAB-KSHV, BC3, BC3-ShControl, BC3-ShHIF1α and BCBL1 cells were maintained in RPMI medium. Human Embryonic Kidney cell line (HEK293T) was obtained from Jon Aster (Brigham and Women’s Hospital, Boston, MA). HEK293T and HEK293-BAC16-KSHV cells were maintained in DMEM medium containing 7% bovine growth serum (BGS) and appropriate antibiotics at 37°C and 5% CO_2_. BC3-ShControl, BC3-ShHIF1α stable cells were maintained in selection media with puromycin (2μg/ml). HEK293T-BAC16-KSHV cells were also maintained in selection with hygromycin (100μg/ml). ShControl, ShHIF1α, pBS-puroA (KSHV terminal repeats), pBS-puroH (6kb KSHV genomic fragment; co-ordinate 26937–33194), pBS-puro-GA5 (10kb KSHV genomic fragment; co-ordinate 36883–47193) and Supercos1-GB22 (15kb KSHV genomic fragment; co-ordinate 85820–100784), pA3F-LANA and pEF-RTA plasmids were described in earlier publications [25]. Generation and maintenance of BC3ShControl, ShHIF1α and ShLANA cells was described earlier [26]. pLVX-ACGFP-vFLIP, pLVX-ACGFP-vCyclin and pLVX-ACGFP-vGPCR constructs were generated by PCR amplification and ligated into Xho1/Hind III, EcoR1/BamH1 and EcoR1/Apa1 site, respectively. For vFLIP and vCyclin, cDNA from KSHV positive BC3 cells was used as template while for vGPCR, pCEFL-vGPCR construct (a gift from Enrique A. Mesri; University of Miami Miller School of Medicine, Miami, FL) was used as template for PCR amplification. For Hypoxic induction, cells were grown in 1%O_2_ at 37°C for the indicated time periods. MG132 was procured from Sigma Aldrich (St. Louis, MO) and was used at a final concentration of 5μM.

### RNA isolation, cDNA synthesis, virion preparation, Infection and KSHV copy number calculation

RNA was isolated by standard phenol chloroform extraction. cDNA was synthesized from 2μg of RNA using superscript cDNA synthesis kit (Applied Biosystem Inc., Foster city, CA) according to manufacturer protocol. The sequence of primers used in the study is provided in the S1 Table. KSHV reactivation and purification was performed according to standard protocol described earlier [26]. To isolate KSHV virion DNA, KSHV virions were resuspended and lysed in 200μl of HMW buffer (10 mM Tris, 150 mM NaCl, 1 mM EDTA, 0.5% SDS and 0.5 mg/ml proteinase K. The virion DNA was further extracted using standard phenol chloroform extraction. Copy number calculation from purified KSHV preparation was performed using standard method. KSHV infection was performed at the multiplicity of infection equivalent to 10 in the presence of 20 μg/ml polybrene as described earlier[26]. The number of extracellular KSHV from the cell culture medium was estimated by real-time PCR through the standard curve method. In brief, equal number of cells in equal volume of cell culture medium were grown in normoxic or hypoxic conditions. Viral reactivation was measured by calculating viral copy number in extracellular culture medium. Viral particles from culture medium were concentrated through centrifugation followed by DNA isolation. DNA pellet were dissolved in an equal volume of water. The standard curve was generated using dilutions of plasmid vector containing KSHV genomic region (15kb KSHV genomic fragment; genome co-ordinates 85820–100784). The sequence of primers used for real-time PCR is given in S1 Table. Unit volume of DNA preparation from extracellular cell culture medium of individual samples were used to estimate the KSHV copy number using primers specific to the cloned KSHV DNA fragment.

### Western blot and confocal microscopy

Protein lysates were separated on 10% polyacrylamide gel followed by wet transfer to nitrocellulose membrane. Skimmed milk (5%) was used for blocking at room temperature for 1 hour with gentle shaking. Primary antibody against CDK2, Cyclin D1, Cyclin E, PDK1, ORC1, ORC2, ORC3, ORC4, ORC5, ORC6, MCM3, GFP, Ubiquitin and GAPDH (Santa Cruz Inc., Dallas, TX), Myc tag and LANA (purified ascites), DNAPOL1A and Cdt1 (Novus Inc., Centennial, CO), CDC6 and CDC45 (Cell Signaling Technology Inc. Danvers, MA), FLAG (Sigma Aldrich Inc., St. Louis, MO) were incubated overnight at 4°C with gentle shaking followed by washing with TBST. Probing with IR conjugated secondary antibody was performed at room temperature for 1 hour followed by washing with TBST. Membranes were scanned on an Odyssey scanner for detection of signals. A complete list and details of antibodies used are provided in S2 Table.

For confocal microscopy, 25,000 cells were semi-dried on 8-well glass slides followed by fixation in 4% paraformaldehyde. Combined permeabilization and blocking was performed in 1XPBS containing 0.3% Triton X-100 and 5% goat serum followed by washing (5 minutes each) with 1X PBS. Anti-LANA antibody (1:200 dilution) was diluted in 1X PBS containing 1% BSA and 0.3% Triton X-100 and was incubated overnight at 4°C. Slides were washed with 1X PBS followed by incubation with Alexa 448 conjugated anti-mouse secondary antibody. DAPI staining was performed for 15 minutes at room temperature followed by washing and mounting. Images were captured by confocal microscope.

### Immuno-precipitation

Whole cell lysates were pre-cleared with Protein Agarose A/G beads and incubated overnight with indicated antibodies with gentle shaking. This was followed by antibody incubation, and Protein Agarose A/G beads were used to collect the immune complexes. The beads were washed 3 times with 1X PBS and resuspended in 60 μl 2X SDS loading dye. The immuno-precipitated complexes were run against 5% input sample lysate.

### Pulse field gel electrophoresis and Southern blot

Pulse field gel electrophoresis (PFGE) and southern blot were described earlier [25]. Briefly, cells were pulsed with Chlorouridine (CldU, 10 μM) in cell culture medium for 4 hours and harvested by centrifugation. Cells were further resuspended in fresh medium containing Iodouridine (IdU, 10 μM) and again pulsed for 4 hours. At the end of pulsing, cells were pelleted, washed with 1XPBS and resuspended in 0.5ml 1XPBS. An equal volume of cell suspension and 1% InCert agarose were equilibrated at 45°C before mixing and molding in the cast for the preparation of cell embedded agarose plugs. The agarose plugs were digested with Proteinase K and followed by washing in 1XTE buffer pH8 with buffer changes after each 24 hours. Final washing of plugs were done in 1XTE pH8 containing 1mM PMSF. Pme1 was used to digest and linearize KSHV episomal DNA. Cell embedded plugs were fitted with agarose gel casting tray in 0.7% low melt agarose. DNA was separated by Pulse field gel electrophoresis for 36 hours on BioRad Chef DRII system (Bio-Rad Inc. Hercules, CA). Post PFGE, Pme1 digested DNA in agarose gel was depurinated and the gel was rinsed with double distilled water followed by denaturation. The gel was then rinsed with double distilled water followed by neutralization and further rinsed with distilled water and equilibrated in SSPE before alkaline transfer on Nylon membrane. Transferred DNA was subjected to UV cross-linking at 1400 Joules and prehybridization was performed at 42°C in prehybridization buffer. Hybridization probes were prepared by random priming method and hybridization was performed at 49°C in prehybridization buffer devoid of salmon sperm DNA. Membrane was washed with low stringency buffer followed by high stringency buffer. Membranes were wrapped in Saran Wrap and exposed to sensitive plates followed by imaging using a Phosphorimager.

### Single molecule analysis of replicated DNA

#### (a) Agarose digestion, yoyo-1 staining, DNA stretching and hybridization

Individual KSHV DNA molecules were visualized by spreading over silanized glass slides and probing with KSHV specific probes [25]. In brief, the region corresponding to area of Southern blot for KSHV DNA was excised from the gel and washed with TE buffer. The block was pre-incubated with digestion buffer (1X TE8/100 mM NaCl/0.1% β-Mercaptoethanol) at 4°C and 100μl digestion buffer was added at 65°C. Agarose digestion commenced by adding Gelase (Epicenter Inc, Madison, WI) and incubated at 45°C. Yoyo-1 staining of DNA was performed by adding Yoyo-1 and β-mercaptoehanol. Yoyo-1 stained DNA was then stretched over the slide by pipetting through one side of a cover slip placed over slide. Stretching of DNA was monitored by visualization using a fluorescent microscope. The slides were denatured by dipping the slides in denaturing buffer (0.1N NaOH/0.1% β-mercaptoethanol in 70% ethanol) and fixed in buffer (denaturing buffer containing 0.5% glutaraldehyde). Slides were serially washed in 70% ethanol, 95% ethanol and 100% ethanol. Biotinylated hybridization probes corresponding to regions of the KSHV genome (6kb: KSHV co-ordinates 26937–33194; 10kb: 36883–47193 and 15kb: 85820–100784)[64] were prepared by nick translation. Biotinylated probes from each reaction were added to hybridization buffer (40% Formamide/1M NaCl, 1%SDS, 10% dextran sulfate, 5 mM Tris pH7.4, with Salmon sperm DNA and floated into the chamber made using cover slips covering the hybridization area. Hybridization was performed at 37°C in a humidified chamber overnight.

#### (b) Immunostaining and FISH

Cover slips were removed and rinsed in 2X SSC/1% SDS. Non-specific hybridized probes were removed by washing the slides in pre-equilibrated high stringency washing buffer at 45°C. Slides were rinsed in 2X SSC/0.1% NP-40 at room temperature followed by washing in 4X SSC/0.1% NP-40. Blocking was performed by adding blocking buffer at room temperature in a humidified chamber. Cover slips were removed and detection mix (1:15 dilution of Alexa 350 conjugated Neutr-Avidin in blocking buffer) was added for each slide and covered with cover slip. After incubation, cover slips were removed followed by repeated incubation with detection mix in a humidified chamber. Mounting was done by adding Vectashield followed by mounting with coverslip and sealing with nail polish. Slides were kept at 4°C before capturing the images on fluorescent microscope.

### Cell cycle analysis by flow cytometry

Cells were harvested by centrifugation at 1000 rpm for 5 minutes and resuspended in 300μl 1X PBS. 700μL ice cold absolute ethanol (70% final) was added drop wise to the cell while gentle vortexing to avoid clumping of cells. Cells were fixed at 4°C on a rotating shaker for 30 minutes followed by washing in PBS. Cells were resuspended in 200μl 1X PBS and incubated with RNase A for 1 hour at 37°C. Following this, 250μl 1X PBS and 50μl Propidium Iodide (1mg/mL) was added to the cells, mixed and stained for 30 minutes at room temperature. The cells were washed with 1XPBS and resuspended then analyzed on FACS Calibur (Becton Dickinson Inc., San Jose, CA, USA). The acquired data were analyzed using FlowJo software (TreeStar Inc., San Carlos, CA, USA).

## Supporting information

S1 Fig Representative image of cell cycle analysis of BJAB and BJAB-KSHV cells grown under normoxic or hypoxic conditions for the indicated time periods. (TIF)

S2 Fig **(A-F)** Real-time PCR analysis of CDK2, CCNE, CCND2 and CCND3 in BJAB and BJAB-KSHV cells grown under normoxic or hypoxic conditions for indicated time periods.
(TIF)

S3 Fig **(A-D)** Real-time PCR analysis of ORC1, ORC3, ORC4 and ORC5 in BJAB and BJAB-KSHV cells grown under normoxic or hypoxic conditions for indicated time periods. (E) Representative images of Western blot analysis of ORC1, ORC3, ORC4, ORC5 and ORC6 in BJAB and BJAB-KSHV cells grown under normoxic or hypoxic conditions. (F) Representative images of Western blot analysis of ORC1, ORC2, ORC3, ORC4, ORC5, ORC6, Cdt1, MCM3 and GAPDH in HEK293T and HEK293T-BAC16-KSHV cells grown under normoxic or 1% O_2_ induced hypoxic conditions. Cells were grown for 24 hours in normoxic or 1% O_2_ induced hypoxic conditions. Equal amounts of whole cell lysate were used for probing protein levels as indicated. GAPDH served as endogenous control.
(TIF)

S4 Fig KSHV protects replication-associated proteins from hypoxia-dependent degradation by negative regulation of ubiquitination. (A). BJAB or BJAB-KSHV cells were grown in medium containing proteosomal inhibitor MG132 and compared with cells grown in normoxia without MG132. In brief, cells were grown for 24 hours in hypoxic conditions, and MG132 treatment was restricted to only last 12 hours to minimize cytotoxic effect of MG132. The results clearly suggested that presence of MG132 had a protective effect on these proteins from hypoxia-mediated degradation. (B). CDC6 was used to demonstrate a role for LANA in the inhibition of proteosomal degradation under hypoxic conditions. Cells expressing mock or LANA were grown under hypoxic conditions (with or without MG132) followed by immuno-precipitation of CDC6 and western blot with ubiquitin antibody. The results showed that the presence of LANA significantly reduced ubiquitination of CDC6 under hypoxic conditions. (C). Hypoxia induces KSHV reactivation. The cells were grown under normoxic or hypoxic conditions and the relative yield of KSHV was monitored by measuring the number of KSHV molecules present in the extracellular culture medium through standard curve based real-time PCR of KSHV DNA using primers for genomic region 89,751–89,832 co-ordinates.
(TIF)

S1 Table List of primers used for real-time PCR. (DOCX)

S2 Table List and details of antibodies used in this study. (DOCX)
