# Supplementary figures and images for: KSHV-encoded LANA protects the cellular replication machinery from hypoxia induced degradation
Source: PLoS Pathog. 2019 Sep 3;15(9):e1008025. doi: 10.1371/journal.ppat.1008025 (PMC6743784; doi:10.1371/journal.ppat.1008025)

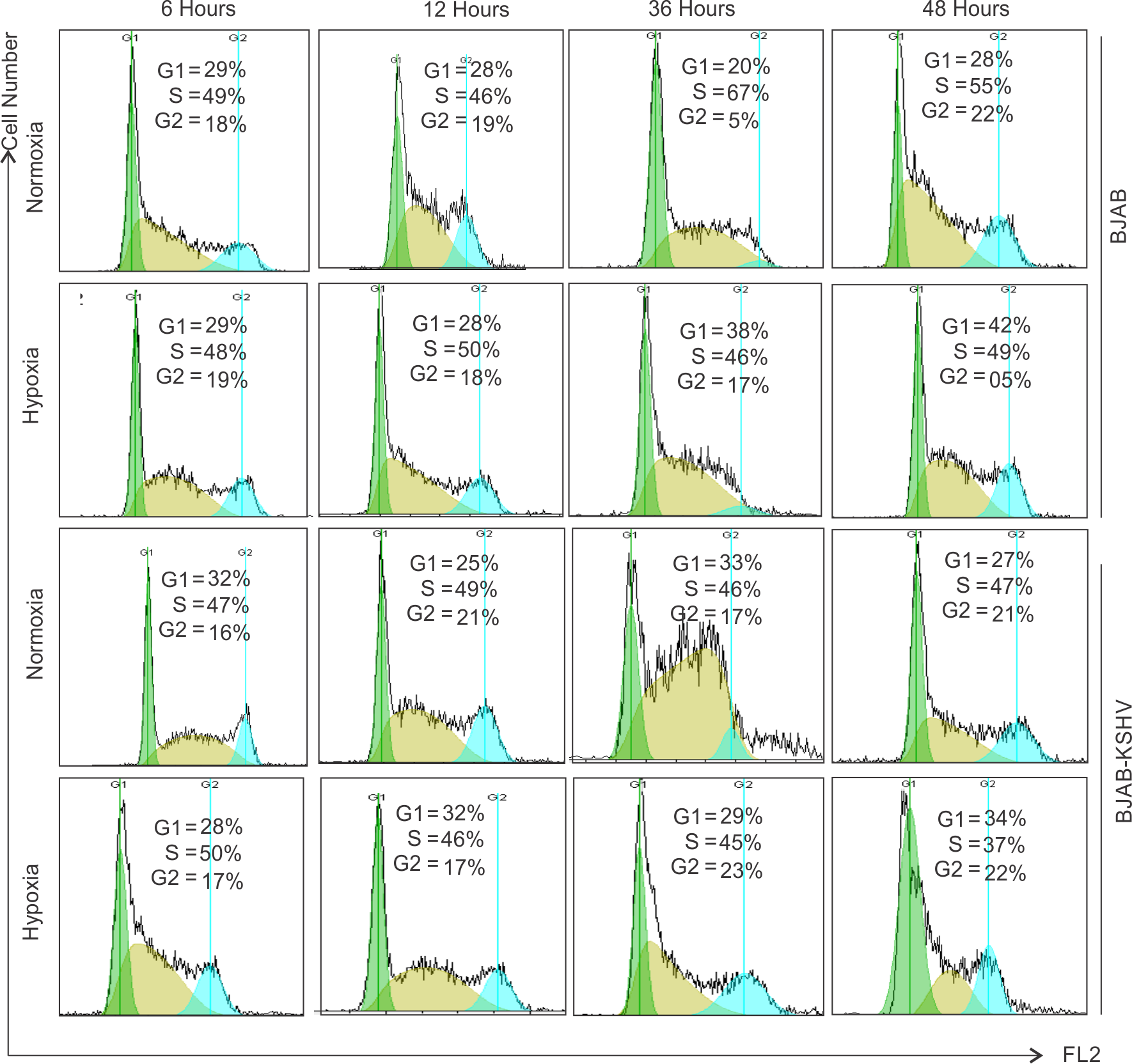

Supplement: S1 Fig — (TIF) [file ppat.1008025.s001.tif]

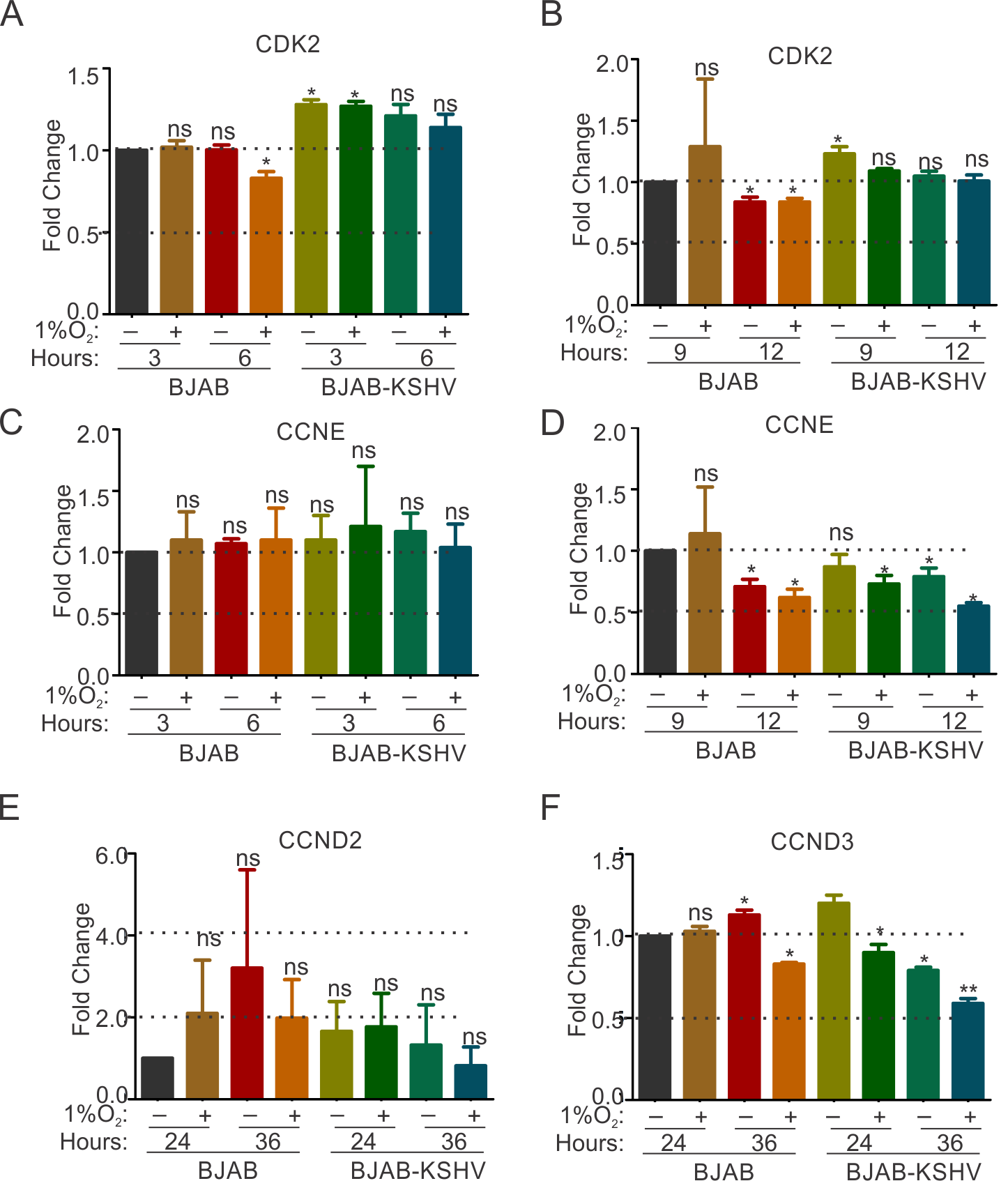

Supplement: S2 Fig — (A-F) Real-time PCR analysis of CDK2, CCNE, CCND2 and CCND3 in BJAB and BJAB-KSHV cells grown under normoxic or hypoxic conditions for indicated time periods. (TIF) [file ppat.1008025.s002.tif]

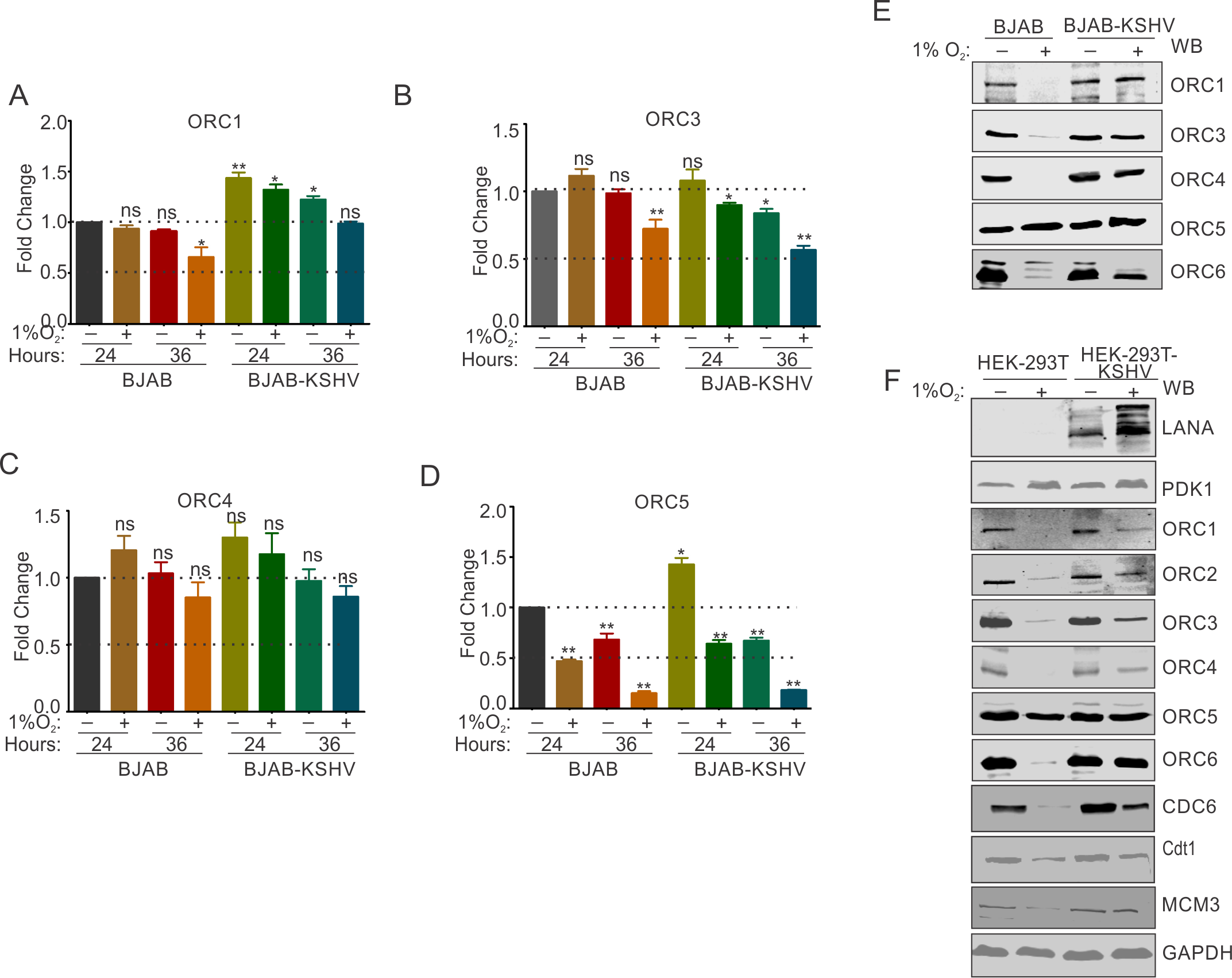

Supplement: S3 Fig — (A-D) Real-time PCR analysis of ORC1, ORC3, ORC4 and ORC5 in BJAB and BJAB-KSHV cells grown under normoxic or hypoxic conditions for indicated time periods. (E) Representative images of Western blot analysis of ORC1, ORC3, ORC4, ORC5 and ORC6 in BJAB and BJAB-KSHV cells grown under normoxic or hypoxic conditions. (F) Representative images of Western blot analysis of ORC1, ORC2, ORC3, ORC4, ORC5, ORC6, Cdt1, MCM3 and GAPDH in HEK293T and HEK293T-BAC16-KSHV cells grown under normoxic or 1% O2 induced hypoxic conditions. Cells were grown for 24 hours in normoxic or 1% O2 induced hypoxic conditions. Equal amounts of whole cell lysate were used for probing protein levels as indicated. GAPDH served as endogenous control. (TIF) [file ppat.1008025.s003.tif]

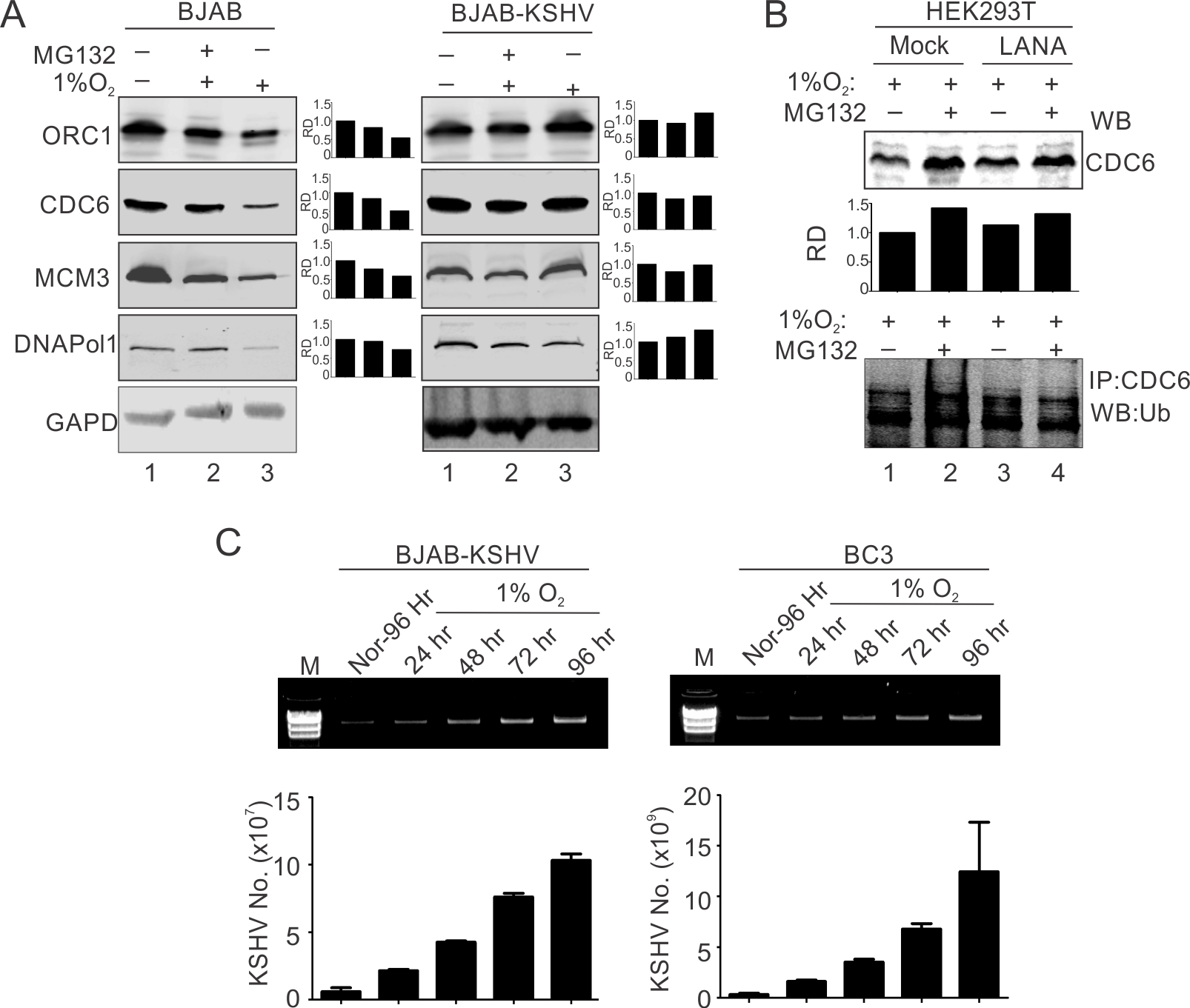

Supplement: S4 Fig — (A). BJAB or BJAB-KSHV cells were grown in medium containing proteosomal inhibitor MG132 and compared with cells grown in normoxia without MG132. In brief, cells were grown for 24 hours in hypoxic conditions, and MG132 treatment was restricted to only last 12 hours to minimize cytotoxic effect of MG132. The results clearly suggested that presence of MG132 had a protective effect on these proteins from hypoxia-mediated degradation. (B). CDC6 was used to demonstrate a role for LANA in the inhibition of proteosomal degradation under hypoxic conditions. Cells expressing mock or LANA were grown under hypoxic conditions (with or without MG132) followed by immuno-precipitation of CDC6 and western blot with ubiquitin antibody. The results showed that the presence of LANA significantly reduced ubiquitination of CDC6 under hypoxic conditions. (C). Hypoxia induces KSHV reactivation. The cells were grown under normoxic or hypoxic conditions and the relative yield of KSHV was monitored by measuring the number of KSHV molecules present in the extracellular culture medium through standard curve based real-time PCR of KSHV DNA using primers for genomic region 89,751–89,832 co-ordinates. (TIF) [file ppat.1008025.s004.tif]
