# Supplementary material for: KSHV-encoded LANA protects the cellular replication machinery from hypoxia induced degradation
Source: PLoS Pathog. 2019 Sep 3;15(9):e1008025. doi: 10.1371/journal.ppat.1008025 (PMC6743784; doi:10.1371/journal.ppat.1008025)
Supplement: S1 Table — (DOCX) [file ppat.1008025.s005.docx]

|  | Forward Primer | Reverse Primer |
| --- | --- | --- |
| ORC1 | AATGGCATGAAGCTGACGGA | GGTGCAGAATTGCTTTGCCA |
| ORC2 | GATGAAGGGGTTGCACAGGA | TGCCAACAGGAGCTGAAACT |
| ORC3 | GTTCCTGGCCCAGTTTCAGA | GGTCTGTCTTCTGAAGCCCC |
| ORC4 | AGCAGTTGCCCAGTGATCTT | ACTGCTATTGGGGTCTGTGC |
| ORC5 | CCAGCAAGAACTGACAAGAGG | GGCCCAAGGAGATGATTGCT |
| MCM3 | AGGTAGTTCTTTGGCAGCGG | TTGTTCAGAAGCCTCGTCGT |
| DNAPOL1A | CGGAGTGTGCACTGGAGAAA | CCTGCAGAACTTTGGGGGTA |
| CDC6 | GCAGTTCAATTCTGTGCCCG | ATAGCTCTCCTGCAAACATCCA |
| CDC45 | AGCCAGAGGGTCCTTCTCTT | CACACTGGAACAAGGCCTGA |
| Cdt1 | CGTCATGCCTGCAACGGGCC | CTCCTCAGGGCGGCCCTCGG |
| CDK2 | GACACGCTGCTGGATGTCA | GAGGGGAAGAGGAATGCCAG |
| CCND1 | TGCGGAAGATCGTCGCCACC | GCTCCAGCGACAGGAAGCGG |
| CCND2 | CACCATCGAGGAGCGCTACC | GACCTCCAGCATCCAGGTGG |
| CCND3 | CCAAGCTGCGCGAGACCACG | CCCTAGGACCAGCACCTCCC |
| CCNE | AGAGGAAGGCAAACGTGACC | TATTGTCCCAAGGCTGGCTC |
| KSHV | CATATCGAACTGTTCTGCCG | TCGCATACAGGGACATGAGC |

Supplementary Table 1: List of primers and their sequence used in this study
