# Supplementary material for: KSHV-encoded LANA protects the cellular replication machinery from hypoxia induced degradation
Source: PLoS Pathog. 2019 Sep 3;15(9):e1008025. doi: 10.1371/journal.ppat.1008025 (PMC6743784; doi:10.1371/journal.ppat.1008025)
Supplement: S2 Table — (DOCX) [file ppat.1008025.s006.docx]

| Antibody | Source |
| --- | --- |
| PDK1 | Santa Cruz Biotechnology (sc-312960) |
| Cyclin D1 | Santa Cruz Biotechnology (sc-4074) |
| Cyclin E | Santa Cruz Biotechnology (sc-377100) |
| CDK2 | Santa Cruz Biotechnology (sc-70829) |
| ORC1 | Santa Cruz Biotechnology (sc-71751) |
| ORC2 | Santa Cruz Biotechnology (sc-32734) |
| ORC3 | Santa Cruz Biotechnology (sc-23888) |
| ORC4 | Santa Cruz Biotechnology (sc-136331) |
| ORC5 | Santa Cruz Biotechnology (sc-20635) |
| ORC6 | Santa Cruz Biotechnology (sc-81646) |
| MCM3 | Santa Cruz Biotechnology (sc-365616) |
| GFP | Santa Cruz Biotechnology (sc-4304) |
| Ubiquitin | Santa Cruz Biotechnology (sc-471120) |
| GAPDH | Santa Cruz Biotechnology (sc-47724) |
| DNAPOL1A | Novus Biologicals (NBP1-47256) |
| CDT1 | Novus Biologicals (NB100-2567) |
| CDC6 | Cell Signaling Technology (3387) |
| CDC45 | Cell Signaling Technology (11881) |
| FLAG | Sigma Aldrich (F1804) |
| Myc-tag | Richard M Longnecker, Northwestern University |
| LANA | Ke Lan, Institut Pasteur of Shanghai |

Supplementary Table 2: List of antibodies used in the study.
